# AUM-302, a novel triple PIM/PI3K/mTOR inhibitor, synergizes with RAS inhibition and impedes the growth of pancreatic ductal adenocarcinoma spheroids and organoids

**DOI:** 10.3389/fphar.2026.1685433

**Published:** 2026-02-11

**Authors:** Emma Wen, Catalina Vera, Sahaana Kesavan, Fatim Kouassi, Joseph F. LaComb, Neecki Zand, David A. Tuveson, Amber N. Habowski, Antonio T. Baines, Agnieszka B. Bialkowska

**Affiliations:** 1 Department of Medicine, Renaissance School of Medicine at Stony Brook University, Stony Brook, NY, United States; 2 Cold Spring Harbor Laboratory, Cold Spring Harbor, NY, United States; 3 Department of Biological & Biomedical Sciences, College of Health & Sciences, North Carolina Central University, Durham, NC, United States; 4 Department of Pharmacology, School of Medicine, the University of North Carolina at Chapel Hill, Chapel Hill, NC, United States

**Keywords:** AUM-302, organoid, pancreatic cancer, PIM, ras, synergy

## Abstract

Pancreatic ductal adenocarcinoma (PDAC) is a significant contributor to cancer-related deaths in the United States. The limited number of well-defined, druggable targets in PDAC has hindered the development of effective treatments. The PIM/PI3K/mTOR pathways, which regulate cell growth, apoptosis, metabolism, and protein synthesis, are often dysregulated in PDAC, leading to various transformed phenotypes, including unchecked cell proliferation. Here, we demonstrate that the triple kinase inhibitor AUM-302 exhibits strong inhibitory efficacy against PDAC growth in 3D formats, including spheroids and organoids. Experiments were conducted using BxPC-3, Capan-2, MIA PaCa-2, and PANC-1 human PDAC cell lines, hF37 2D organoid-derived PDAC cell line, and primary (hF37, hF31, hF44, hT1) and metastatic (hM1a) patient-derived pancreatic cancer organoids. Single- and dual-kinase inhibitors TP-3654, GDC-0941, BEZ-235, respectively, and DMSO were used as controls. The synergy studies were performed using AUM-302 and the RAS inhibitor RMC-6236. Our results showed that AUM-302 significantly inhibited the viability and proliferation of PDAC cell spheroids and organoids more effectively than the controls. The activity of mTOR, AKT, and S6 pathways was decreased as measured by the expression levels of the phosphorylated proteins in hT1 and hM1a organoids after 24 h of AUM-302 treatment, suggesting that AUM-302 reduced the activity of these kinases. Finally, combinatorial assays revealed synergy between AUM-302 and the RAS inhibitor RMC-6236 in reducing the growth of hT1 and hM1A organoids. By blocking kinase activity, AUM-302 demonstrates potent inhibition in PDAC cell lines and organoids across two 3D culture formats. Treatment with this novel triple PIM/PI3K/mTOR inhibitor may also chemosensitize PDAC to other cancer therapies, such as RAS inhibitors.

## 1 Introduction

Pancreatic ductal adenocarcinoma (PDAC) is the most prevalent type of pancreatic cancer, with a 5-year survival rate of around 8% due to late disease diagnosis and ineffective treatment options (Blackford et al., 2024; Siegel et al., 2025; Stoop et al., 2025). The majority of PDAC is recognized in the advanced and metastatic stage when surgery and radiation therapy are futile, and current chemotherapy and targeted therapies still fail to ensure long-term survival (Meneses-Medina et al., 2022; Li et al., 2024). However, a deeper understanding of PDAC pathophysiology, combined with thorough analysis of specimens via whole-genome or whole-exome sequencing, has enabled the identification of pathways and proteins that can be targeted in the development of therapies. The majority of inhibitors approved or being tested target *KRAS* or the RAS family, *PARP*, *SMAD4*, *RET*, *NTRK*, and components of Tyrosine kinase pathways (Leroux and Konstantinidou, 2021; Li et al., 2024). These therapies are being combined with standard-of-care treatments such as gemcitabine, checkpoint inhibitors, and immunotherapies (Burris et al., 2023). Unfortunately, despite numerous scientific advancements in single agents and combinatorial therapies for PDAC, little progress has been made to improve patients’ quality of life and survival (Hajatdoost et al., 2018; Wang et al., 2024; Stoop et al., 2025).

The PI3K/AKT/mTOR pathway is one of the major drivers of PDAC (Murthy et al., 2018). Its dysregulation is caused by mutations in *AKT2*, *PIK3CA*, or *PTEN*, which integrate extracellular and intracellular signals that drive cancer growth, progression, metastasis, and survival (Conway et al., 2019). Notably, the PI3K/AKT/mTOR pathway converges with other crucial pathways that are upregulated in PDAC, including the MAPK/EGFR pathway, with *KRAS* as a major effector, which is mutated in more than 90% of PDAC cases (Murthy et al., 2018; McIntyre et al., 2024; Yousef et al., 2025). The most frequent KRAS mutations are G12D, G12V, and G12R, with Q61H being less frequent (Zhang et al., 2023). As these mutations enhance KRAS’s ability to bind GTP and activate downstream proteins, targeting mutated KRAS has become a promising approach most recently. Two KRAS inhibitors targeting G12C mutations, sotorasib, and adagrasib, were approved by the FDA in 2021 and 2022, respectively, although given the rarity of PDAC G12C mutations this has little benefit for PDAC patients (Ostrem et al., 2013; Stickler et al., 2024). However, since then, single and combinatorial therapies, including pan-RAS inhibitors and G12D-specific inhibitors, have entered clinical trials. One of them is RMC-6236, an RAS-MULTI(ON) inhibitor that, by design, binds to the active, GTP-bound state of the RAS protein and has the potential to inhibit multiple KRAS mutants (Jiang et al., 2024). Currently, RMC-6236 has entered phase III of clinical trials for PDAC, where it will be combined with standard treatments for gastrointestinal cancers to evaluate its long-term efficacy.

The PIM kinase family, consisting of PIM1, PIM2, and PIM3, are serine-threonine kinases that have been shown to regulate proliferation, apoptosis, metabolism, stem cell function, angiogenesis, tumorigenesis, and radioresistance (Brault et al., 2010; Xu et al., 2011; Wang et al., 2013; Li and Mukaida, 2014; Xu et al., 2014; Chang et al., 2016; Xu et al., 2016; Choudhury et al., 2024). Notably, PIM1 and PIM3 levels are increased in PDAC tumors compared to adjacent normal tissue and are inversely correlated with patient survival (Li et al., 2006; Xu et al., 2011; Blanco et al., 2016; Xu et al., 2016). Studies have shown that PDAC-associated hypoxia increases PIM levels and underlies PIMs’ role in chemoresistance (Chen et al., 2009a; Chen et al., 2009b; Blanco et al., 2016; Casillas et al., 2018; Toth et al., 2022; Choudhury et al., 2024). It has also been demonstrated that genetic or chemical inactivation of PIM1 or PIM3 *in vitro* and *in vivo* led to inhibition of PDAC development and progression and decreased chemoresistance (Li et al., 2006; Chen et al., 2009b; Mukaida et al., 2011; Xu et al., 2013; Liang et al., 2015; Nakano et al., 2015; Panchal and Sabina, 2020). As such, PIM kinases can serve as a biomarker and a potential molecular target for anti-cancer therapy (Panchal and Sabina, 2020; Atalay and Ozpolat, 2024; Chen et al., 2024).

Our previous study demonstrated that AUM-302, a triple kinase inhibitor (PIM/PI3K/mTOR), significantly reduced cell proliferation and dysregulated the cell cycle in pancreatic cancer cell lines by modulating several key signaling pathways (Ingle et al., 2023). In the current manuscript, we focused on a three-dimensional growth system of human pancreatic cancer cells as well as human pancreatic cancer organoids to assess the efficacy of AUM-302, both alone and in combination with other treatments. It has been shown that three-dimensional models such as PDAC spheroids, and especially PDAC organoids, better recapitulate characteristics of human tumor growth (Boj et al., 2015; Tiriac et al., 2018; Demyan et al., 2022; Le Compte et al., 2023; Habowski et al., 2024). Here, we show that AUM-302 efficiently inhibits the growth of PDAC spheroids and organoids compared to single and dual PI3K/mTOR inhibitors. Importantly, we demonstrate in the settings of PDAC organoids that AUM-302 synergizes with the RAS inhibitor RMC-6236 to impede their growth. Analysis of the downstream targets PIM/PI3K/mTOR and KRAS pathways in the combinatorial treatment showed downregulation of pERK1/2 and pS6 activity compared to the single treatments. In summary, we demonstrated that AUM-302 inhibitory activity is preserved in spheroid and organoid models, and it can synergize with currently clinically tested RAS inhibitors.

## 2 Materials and methods

### 2.1 Cell lines, organoids, and compounds

PDAC cell lines BxPC-3 (CRL-1687), Capan-2 (HTB-80), MIA PaCa-2 (CRL-1420), and PANC-1 (CRL-1469) were purchased from ATCC (Manassas, VA). BxPC-3 cells were maintained in RPMI-1640 medium, and Capan-2 cells in McCoy’s medium. MIA PaCa-2 and PANC-1 cells were maintained in the DMEM medium. All media were supplemented with 10% FBS and 1% penicillin/streptomycin. PDAC organoids hF31, hF37, hF44, hT1, and hM1a were obtained from the Stony Brook Cancer Center Biobank and the Cold Spring Harbor Laboratory Biobank and grown according to previous publications (Huch et al., 2013; Boj et al., 2015). All cell lines and organoids were maintained at 37 °C and 5% CO_2_. All experiments were performed on cell lines with a passage range between 5 and 29. BEZ235, GDC-0941, TP-3654, RMC-6236, and gemcitabine were purchased from Selleck Chemicals (Houston, TX), and AUM Biosciences generously provided AUM-302. Another vial of RMC-6236 was purchased from ChemieTek (Indianapolis, IN). BEZ235, GDC-0941, TP-3654, AUM-302, RMC-6236, and gemcitabine were suspended in DMSO.

### 2.2 Cell viability assay

#### 2.2.1 Spheroids

BxPC-3, Capan-2, MIA PaCa-2, PANC-1, and hF37 2D cell lines were seeded at 3 × 10^3^ cells per well in a 96-well round-bottom ultra-low attachment surface spheroid microplate (Corning) in 50 μL of appropriate media and incubated for 24 h. Following incubation, the growth media were supplemented with 50 μL vehicle control (0.1% DMSO) or various concentrations of BEZ235, GDC-0941, TP-3654, and AUM-302. Images of the spheroids were taken 24-, 48-, and 72 h post-treatment using a Nikon ECLIPSE Ti2 inverted microscope. Cell viability was assessed 72 h after the treatment using 3D Cell Titer-Glo (Promega) and measured on the SpectraMax® M3 Microplate Reader. The IC_50_ and viability values were calculated using GraphPad Prism for Windows version 10.4.1 (GraphPad Software).

#### 2.2.2 Organoids–single-agent

hF31, hF37, hF44, hT1, and hM1a PDAC organoids were seeded in Matrigel (50% Matrigel:50%dPBS) in a 96-well microplate (Corning) in 100 μL of human pancreatic organoid media. The growth media were supplemented with 100 μL vehicle control (0.1% DMSO) or various concentrations of BEZ235, GDC-0941, TP-3654, and AUM-302. Images of the organoids were taken 24-, 48-, and 72 h post-treatment using a Nikon ECLIPSE Ti2 inverted microscope. The cell viability was assessed 72 h after the treatment using 3D Cell Titer-Glo (Promega) and measured on the SpectraMax® M3 Microplate Reader. The values were calculated using GraphPad Prism for Windows version 10.4.1 (GraphPad Software).

#### 2.2.3 Organoids–combinatorial treatment

hM1a and hT1 organoids were seeded in Matrigel in a 6-well cell repellent plate with 3 mL of human pancreatic organoid media. Following incubation, the organoids were treated for 24 h prior to protein lysate harvest. The Matrigel domes containing the organoids were washed with dPBS and subsequently harvested in Matrigel Melting Solution (dPBS, 1x HEPES, 1x GlutaMAX, 12 mM EDTA, 12 mM EGTA, and 4,5 g/L Dextrose) with PhosSTOP™ (Roche) and cOmplete™, Mini, EDTA-free Protease Inhibitor Cocktail (Roche) (MMS-PPI). After the Matrigel had melted and was removed, the organoids were resuspended in RIPA-PPI, and the protein lysates were collected after incubation on ice and centrifuging.

#### 2.2.4 Synergy analysis

Synergy assays were conducted similarly to the viability assay. Each point (mean ± standard error of the mean) represents the growth of compound-treated cells compared to vehicle-only-treated cells. The expected growth inhibition was calculated using the HSA independence model and overlaid on the experimental growth curve in GraphPad Prism for Windows version 10.4.1. The HSA model assumes that in the absence of interaction between tested compounds, the combination treatment should not reach the effect of the more potent single drug at any tested dose (Lehar et al., 2007). The synergy analysis was performed using SynergyFinder 3.0 in R (Ianevski et al., 2022; Zheng et al., 2022). The data are presented as a dose-response matrix (mean value of growth inhibition ±SD, synergy scores, and a synergy barometer (expected and observed inhibition for a given dose combination). The synergy scores below −10 are designated as an antagonistic interaction between the tested compounds, between −10 and 10 as likely to be additive, and above 10 as possible to be synergistic.

#### 2.2.5 Western blot analysis

The hT1 and hM1a PDAC organoids were seeded in Matrigel in a 6-well plate (Corning) in 3 mL of appropriate media and incubated for 24–48 h. Following incubation, the growth media were supplemented with 0.2% DMSO or various concentrations of AUM-302 and RMC-6236. Twenty-four- or 48-h post-treatment, organoids were washed with ice-cold dPBS and lysed in Laemmli buffer, and protein extracts were subjected to electrophoresis in 4%–20% gels. The proteins were then transferred to a nitrocellulose membrane, blocked in 5% non-fat milk in 1 x TTBS buffer, and probed overnight with the following primary antibodies against: p-mTOR (Cell Signaling, #2971), mTOR (Cell Signaling, #2972), p-β-catenin (Cell Signaling, #9566), β-catenin (Cell Signaling, #8480), pSTAT3 (Cell Signaling, #9145), STAT3 (Cell Signaling, #30835), p-p70S6 (Cell Signaling, #9208), p70S6 (Cell Signaling, #9202), p-NFκB (Cell Signaling, #3033), NFκB (Cell Signaling, #4764), pAKT (Cell Signaling, #9271), AKT (Cell Signaling, #9272), c-Myc (Cell Signaling, #9402), pERK1/2 (Cell Signaling, #9101), ERK1/2 (Cell Signaling, #9102), p-p38 (Cell Signaling, #9215), p38 (Cell Signaling, #9219), p-pS6 (Cell Signaling, #4856), pS6 (Cell Signaling, #2317), and β-actin (Millipore-Sigma, #A1978), and with the following secondary antibodies HRP-conjugated for 1 hour: goat anti-rabbit (VWR, #111-035–144) and goat anti-mouse (Millipore-Sigma, #AP200P). Protein bands were detected using Pierce™ SuperSignal™ West Pico PLUS Chemiluminescent Substrate (VWR, #34580) using Azure 400 (Azure Biosystems). Densitometry analysis was performed using FIJI software (Schindelin et al., 2012).

### 2.3 Statistical analysis

The analysis was performed using an appropriate statistical test with a value of p < 0.05 considered significant. This analysis was performed using GraphPad Prism for Windows version 10.4.1 (GraphPad Software).

## 3 Results

AUM-302 inhibits the growth of human pancreatic cancer spheroids.

**FIGURE 1:**
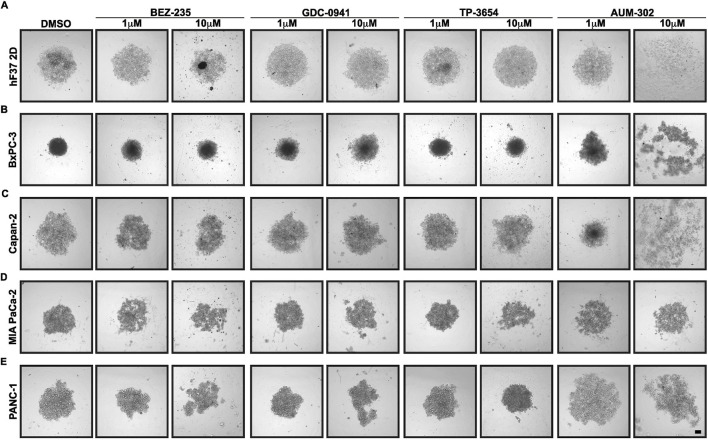
AUM-302 exerts a more substantial inhibitory growth effect in PDAC spheroids than other tested compounds. **(A)** hF37 PDAC organoid-derived 2D cell line, **(B)** BxPC-3, **(C)** Capan-2, **(D)** MIA PaCa-2, and **(E)** PANC-1 PDAC cell lines were treated as spheroids with DMSO (vehicle) and 1 μM and 10 μM concentrations of BEZ-235, GDC-0941, TP-3654, or AUM-302 for 72 h. The figure displays representative images from a sample of *N = 5*. The scale bar depicts 100 μm.

To determine the effects of AUM-302 and previously tested PI3K and mTOR inhibitors, we used ultra-low attachment plates that support the growth of cell lines and organoids as spheroids in a three-dimensional setting. We seeded three human pancreatic cancer cell lines (BxPC-3, MIA PaCa-2, and PANC-1), treated with DMSO (vehicle) or various concentrations (between 1 nM and 1 μM) of BEZ-235 (a dual ATP-competitive PI3K and mTOR inhibitor), GDC-0941 (a potent inhibitor of PI3Kα/δ), TP-3654 (a second-generation PIM inhibitor), and AUM-302 (a triple PIM/PI3K/mTOR inhibitor) over a 3-day period and imaged them every 24 h (Supplementary Figures 1–9). The analysis using a limited concentration range of compounds showed that BEZ-235 and AUM-302 demonstrated greater potential to inhibit spheroid growth than GDC-0941 and TP-3654. As such, we extended the treatment range and, in addition, included the Capan-2 PDAC cell line and a 2D derivative of the human pancreatic cancer organoid line (hF37) in the spheroid system, and performed a 3-day treatment to assess spheroid viability. Using the 2D derivative of the human pancreatic cancer organoid line will enable initial screening and scalable analysis before advancing to more resource-intensive 3D Matrigel organoid culture. After 72 h of treatment, representative images showed that AUM-302 exhibited the most significant inhibitory effect on spheroid growth among the tested compounds (Figure 1; Supplementary Figures 10–14). Of the four tested compounds, GDC-0941 and TP-3654 were shown to be the least effective in reducing the growth of spheroid cultures as even the 10 μM concentrations of these drugs did not affect the growth except GDC-0941-treated Bx-PC3 cells (Figure 1; Supplementary Figures 10–14). These results align well with our previous data, which showed that these compounds were the least effective at inhibiting the proliferation of PDAC cell lines.

**FIGURE 2:**
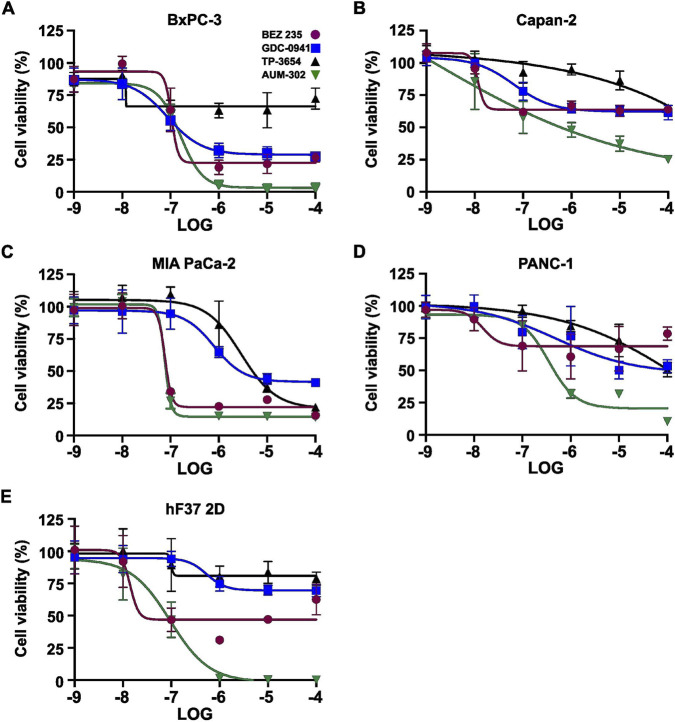
BEZ-235, GDC-0941, TP-3654, and AUM-302 inhibit the viability of multiple pancreatic cancer cell lines in a spheroid setting. Pancreatic cancer cell lines BxPC-3 **(A)** Capan-2 **(B)** MIA PaCa-2 **(C)** PANC-1 **(D)** and organoid-derived cell line hF37 2D **(E)** were treated with variable concentrations of BEZ-235 (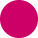) GDC-0941(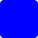) TP-3654(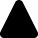) and AUM-302 (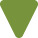) for 72 h, and cell viability was measured using 3D Cell Titer-Glo. The results are shown as mean ± SD (*N = 5*) and normalized to the control (DMSO). The LOG -9 represents 1 nM, -8 – 10 nM, -7 – 100nM, -6 – 1 μM, -5 – 10 μM, -4 – 100 μM.

In contrast, BEZ-235 and AUM-302 were the most efficacious in reducing the growth of spheroids, with AUM-302 being superior. AUM-302 concentrations of 10 nM caused visible shrinkage of hF37, Bx-PC3, and Capan-2, while BEZ-235 had little or no effect (Supplementary Figures 10–12). Conversely, the higher concentration of AUM-302, such as 1 μM or 10 μM, caused considerably more death of spheroids than BEZ-235, as shown with hF37 2D, Bx-PC3, Capan-2, MIA PaCa-2, and PANC-1 (Figure 1). These changes in spheroid compactness, loss of structure, and the cell viability assay results confirm that AUM-302 is a significantly more potent compound inducing cell death in all tested lines grown as spheroids, providing a deeper understanding of its efficacy (Figure 2; Table 1). These observations also agree with our previous results using 2D PDAC cell lines.

**TABLE 1:** IC_50_ values of BxPC-3, Capan-2, MIA PaCa-2, PANC-1, and hF37 2D treated with BEZ-235, GDC-0941, TP-3654, and AUM-302 in the setting of spheroids.

Cell line	BEZ-235	GDC-0941	TP-3654	AUM-302
BxPC-3	4.874E-07	8.677E-08	Unstable	1.608E-07
Capan-2	1.164E-08	6.43E-08	1.44E+09	1.125E-09
MIA PaCa-2	7.686E-08	7.981E-07	2.92E-06	7.626E-08
PANC-1	1.516E-08	4.874E-07	2.43E-04	3.549E-07
hF37 2D	1.399E-08	5.425E-07	9.977E-08	9.462E-08

### 3.1 AUM-302 significantly inhibits the growth of human pancreatic cancer organoids

**FIGURE 3:**
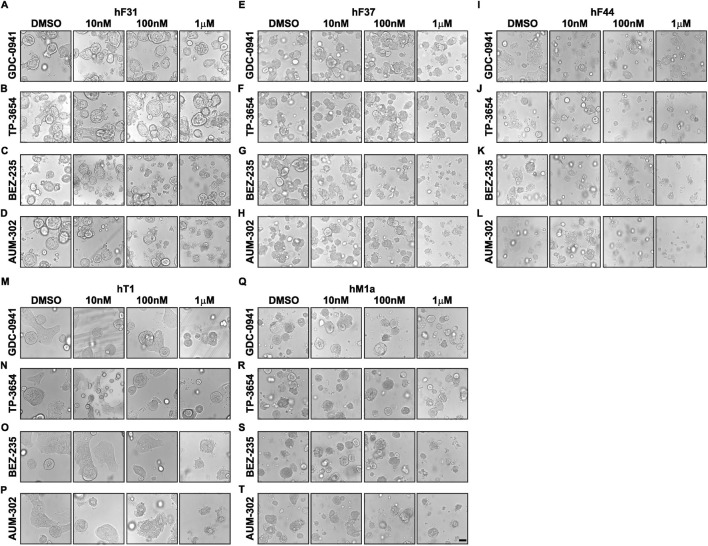
AUM-302 exerts a more substantial inhibitory growth effect in hF31, hF37, hF44, hT1, and hM1a PDAC organoids than other tested compounds. hF31 **(A–D)** hF37 **(E–H)** hF44 **(I–L)** hT1 **(M–P)** and hM1a **(Q–T)** PDAC organoids were seeded in Matrigel and treated with DMSO (vehicle) and 10 nM, 100 nM, and 1 μM concentrations of GDC-0941 **(A,E,I,M,Q)** TP-3654 **(B,F,J,N,R)** BEZ-235 **(C,G,K,O,S)** or AUM-302 **(D,H,L,P,T)** for 72 h. The figure displays representative images from a sample of *N = 5*. The scale bar depicts 100 μm.

**FIGURE 4:**
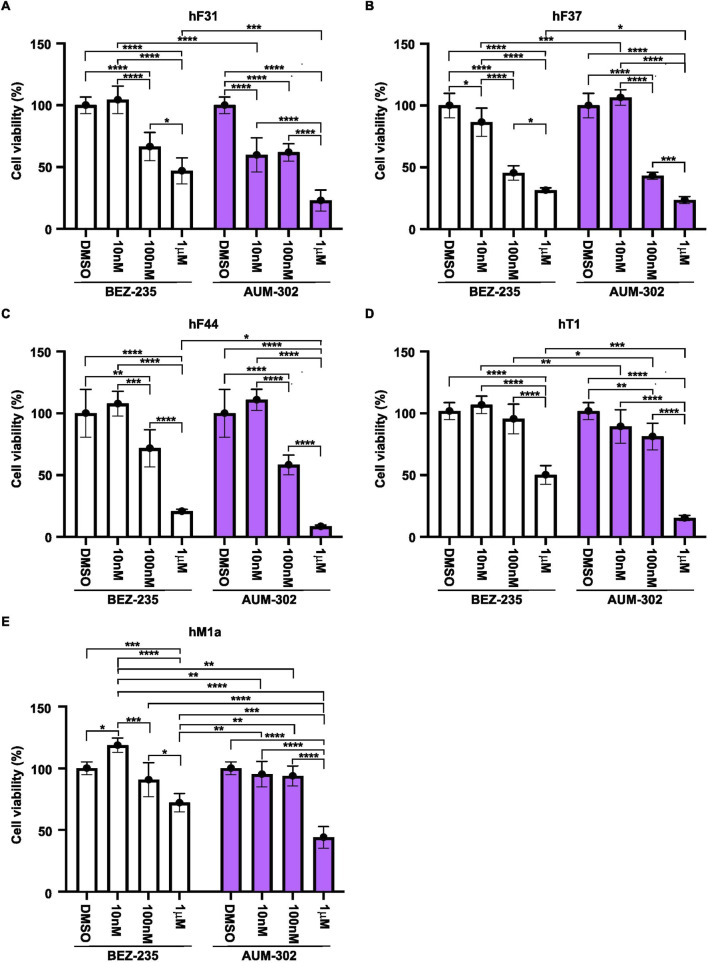
AUM-302 significantly exerts a stronger inhibitory growth effect on PDAC organoids than BEZ-235. hF31 **(A)** hF37 **(B)** hF44 **(C)** hT1 **(D)** and hM1a **(E)** PDAC organoids were seeded in Matrigel and treated with DMSO (vehicle) and 10 nM, 100 nM, and 1 μM concentrations of BEZ-235 and AUM-302 for 72 h. At the end of the incubation, cell viability was measured using 3D Cell Titer-Glo. The results are shown as mean ± SD (*N = 5*) and normalized to the control (DMSO). The statistical analysis was performed using a two-way ANOVA in GraphPad Prism version 10.4.1. *p < 0.05, **p < 0.01, ***p < 0.001, and ****p < 0.0001.

We tested four human pancreatic cancer organoids to assess the efficacy of BEZ-235, GDC-0941, TP-3654, and AUM-302 on their viability. PDAC organoid lines hF31, hF37, hF44, hT1, and hM1a were seeded in Matrigel domes, treated with DMSO (vehicle), BEZ-235, GDC-0941, TP-3654, and AUM-302 at 10nM, 100 nM, and 1 μM concentrations for 72 h, and imaged. Our results demonstrate that the GDC-0941 and TP-3654 compounds had minimal effects on the growth of the four tested organoid lines (Figure 3; Supplementary Figure 15), resulting in shrinkage and disintegration of the organoids, only at higher compound concentrations and, as such, demonstrating little to no inhibitory effects. For example, there was a negligible difference on hF31, hT1, and hM1a even at 1 μM concentrations of GDC-0941 and TP-3654 compounds. In contrast, BEZ-235 and AUM-302 affected the growth of the organoids at lower compound concentrations, and treatment with AUM-302 was more deleterious to organoid growth than the BEZ-235 treatment (Figure 3). The 3D Cell Titer-Glo analysis confirmed observed changes in the organoids’ structure (Figure 4; Supplementary Figure 16). GDC-0941 and TP-3654 also have minimal to no effect on the growth of tested PDAC organoids (Supplementary Figure 16). The GDC-0941 and TP-3654 compounds showed minimal effects on the growth of hF31, hF37, hF44, hT1, and hM1a even at the 1 μM concentration. In contrast, BEZ-235 and AUM-302 significantly reduced the growth of the PDAC organoids even at a lower concentration of 100 nM. As such 100 nM of BEZ-235 resulted in 33.39% ± 11.5, 54.55% ± 5.9, 28.28% ± 14.9, 4.56% ± 11.9, and 9.3% ± 13.8, while AUM-302 in 33.12% ± 7.1, 56.79% ± 2.9, 41.7% ± 7.95, 18.84% ± 10.8, and 6.2% ± 8.1 of growth inhibition in hF31, hF37, hF44, hT1, and hM1a respectively. The treatment with the higher concentration of 1 μM BEZ-235 and AUM-302 demonstrated the superior potency of the latter. The 1 μM BEZ-235 treatment resulted in 53.02% ± 10.6, 68.58% ± 2.03, 79.35% ± 1.7, 49.87% ± 7.6, and 27.9% ± 7.4, while with AUM-302 in 77% ± 8.5, 76.46% ± 2.9, 91.56% ± 1.2, 84.7% ± 2.1, and 56% ± 8.7 in hF31, hF37, hF44, hT1, and hM1a respectively. This comparison demonstrated that AUM-302 has substantial inhibitory potential on the growth of PDAC organoids compared to BEZ-235 (Figure 4).

Because the AUM-302 compound showed the most significant inhibitory potential in PDAC organoids, we assessed the activity of signaling pathways previously shown to be affected by its treatment. Initially, we treated two PDAC organoids with DMSO, 10nM and 100 nM concentrations of AUM-302 for 24 h. We assessed the activity of various pathways within a day of treatment to determine the changes that caused the growth inhibition. In the case of both treated organoids, the changes were visible after treatment with 100 nM AUM-302 (Supplementary Figure 17). We observed modifications of the levels of phospho-mTOR (Ser2448), phospho-AKT (Ser473), and phospho-S6 (Ser235/236), suggesting decrease in the activity of PIM//PI3K/mTOR pathways. In contrast, β-catenin, STAT3, NFκB, ERK, c-Myc, p70, and p38 pathways were not primarily affected under the tested conditions. To more thoroughly assess the changes to the signaling pathways’ activity we expanded the experiment and tested all five organoids over 2 days at 10nM, 100 nM, and 1 μM concentrations of AUM-302, alongside the control (DMSO) (Supplementary Figures 18-22). We have noticed changes in various pathway activities; however, the responses were heterogeneous across different PDAC organoids. We analyzed total protein levels and, when feasible, their phosphorylated counterparts. As a whole, treatment with 1 μM AUM-302 for 2 days resulted in a loss of protein expression, which is reflected in reduced viability, as shown in Figure 4, and reduced levels of all proteins with the loading control–β-actin (Supplementary Figures 18-22). To varying degrees, we observed reductions in c-Myc protein levels, mTOR, AKT, and S6, as well as in the levels of phosphorylated mTOR, AKT, and S6 upon treatment with 10nM and 100 nM of AUM-302 at 24 and 48 h. These results could reflect the heterogeneity of the tested organoids.

### 3.2 AUM-302 effectively synergizes with a pan-RAS inhibitor to suppress the growth of human PDAC organoids

Here, we tested the likelihood of synergy between gemcitabine, a nucleoside analog inhibiting DNA synthesis, RMC-6236, and AUM-302 in suppressing PDAC organoids growth. The synergy analysis showed that gemcitabine and AUM-302, by and large, do not synergize in hT1 and hM1a organoids under tested conditions (Supplementary Figures 23, 24). The cell viability (Supplementary Figures 23A-24A) results were employed to calculate HSA Synergy Scores and presented as a matrix (Supplementary Figures 23B, 24B) and depicted as HSA plots (Supplementary Figures 23C, 24C). Overall, gemcitabine and AUM-302 compounds were a potent combination, but likely only additive. In hT1 organoids, the positive HSA Synergy Score was calculated for only three compound combinations, while in hM1a organoids, the HSA Synergy Scores under five conditions emerged as synergistic for gemcitabine and AUM-302. We used the Synergy Barometer to calculate the expected versus observed cell viability and determine whether it resulted in significant inhibition. In essence, we used the combination with the highest calculated synergy score for each of the organoid. The observed inhibitions of the cell viability in hT1 and hM1a organoids were barely above the expected viability determined by four synergy algorithms (Bliss, HAS, ZIP, and Loewe) (Supplementary Figures 23D, 24D). As such, in hT1 and hM1a, treatment with gemcitabine and AUM-302 did not result in synergistic inhibitions of proliferation.

**FIGURE 5:**
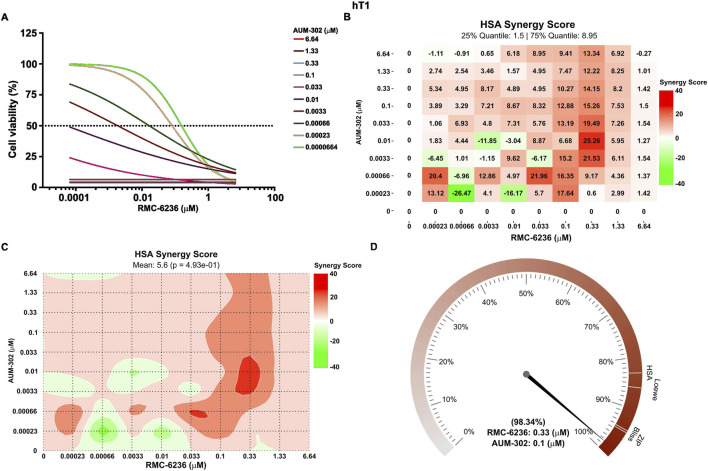
AUM-302 synergizes with RMC-6236 to inhibit the growth of hT1 PDAC organoids. hT1 PDAC organoids were seeded in Matrigel and treated with various concentrations of AUM-302, RMC-6236, and a combination of both compounds. Seventy-two hours post-treatment, the cell viability was assessed using 3D Cell Titer-Glo and analyzed using a non-linear regression model of log(inhibitor) vs. response–variable slope (four parameters) equation in GraphPad Prism version 10.4.1. and Synergy Finder. **(A)** Cell viability of mono-and combinatorial treatment. The results are shown as mean ± SD (*N = 5*) and normalized to the control (DMSO). **(B)** The HSA Synergy Score is a dose matrix inhibition response. **(C)** The HSA Synergy Score is shown as a plot contour. **(D)** The graph was generated using a Plot Barometer using viability results. The needle points to the observed growth inhibition, denoted in percentage values, for the synergy between AUM-302 0.1 μM and RMC-6236 0.33 μM. The four white lines represent the predicted inhibition values calculated by the HSA, Loewe, ZIP, and BLISS models at specific concentrations.

In contrast, the treatment of RMC-6236 and AUM-302 yielded numerous combinations of synergistic outcomes (Figure 5; Supplementary Figure 25), as shown by the cell viability data and calculated HSA Synergy Score. As mentioned above, we used the Barometer function to determine the expected and observed response value for the highest HSA synergy score. For hT1, RMC-6236 0.33 μM and AUM-302 0.1 μM yielded almost 99% growth inhibition compared to the expected values between 83%-96% (Figure 5D). In the case of the hM1A, the treatment of RMC-6236 at 0.33 μM and AUM-302 at 0.01 μM yielded 100% inhibition of growth compared to the expected values between 96%-98% (Supplementary Figure 25D). These data showed the likelihood of these two compounds synergizing to induce cell deaths in PDAC organoids.

### 3.3 Treatment with AUM-302 and RMC-6236 results in the modification of the activity of crucial signaling pathways in PDAC organoids

**FIGURE 6:**
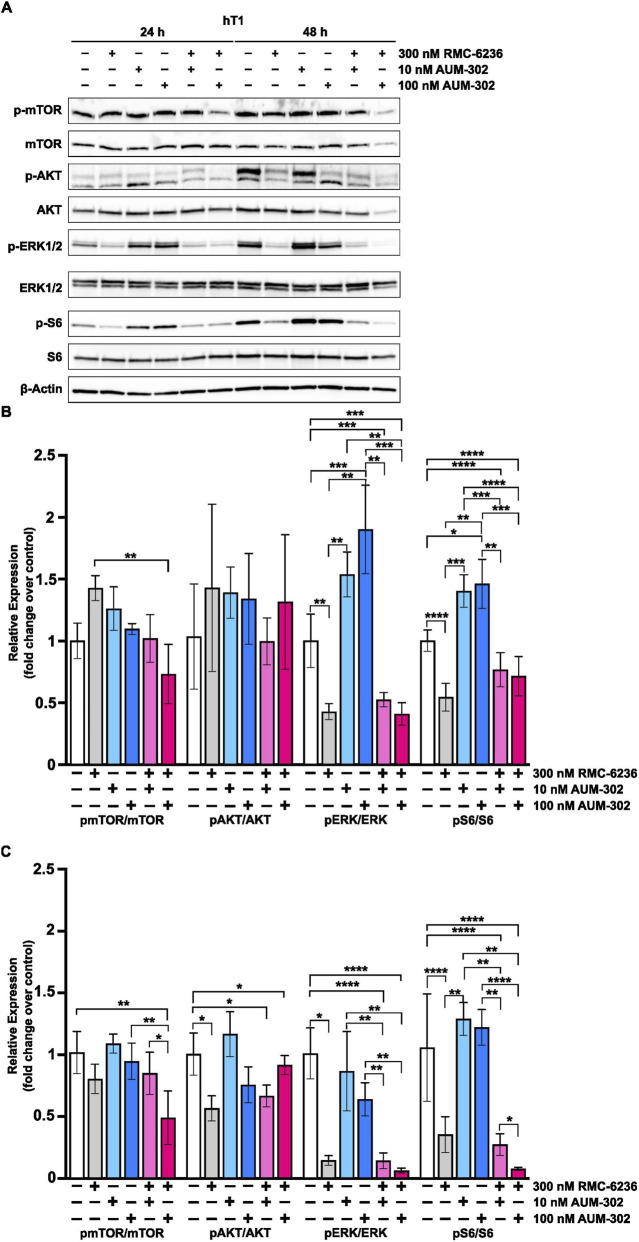
AUM-302 and RMC-6236 affected the activity of the signaling pathways in the hT1 PDAC organoids. hT1 organoids were treated with DMSO (vehicle), 300nM RMC-6236, 10 nM and 100 nM AUM-302, and combinations of thereof for 24 and 48 h. **(A)** Western blots were performed for mTOR, AKT, ERK, S6, phosphorylated counterparts, and β-Actin as a loading control. **(B,C)** Densitometry analysis was conducted to quantify relative protein expression, normalized to β-actin control and non-phosphorylated proteins in samples after 24- and 48-h post-treatment. Densitometry was measured with ImageJ and analyzed with GraphPad Prism as described in “Materials and Methods.” Data are presented as mean ± SD values (*N* = 3). *, *P* < 0.05; **, *P* < 0.01; ***, *P* < 0.001; ****, *P* < 0.0001.

To further characterize the synergy between RMC-6236 and AUM-302, we treated the hT1 PDAC organoid with DMSO (vehicle), 300 nM of RMC-6236, 10 nM of AUM-302, and 100 nM of AUM-302 and a combination of them and collected protein extracts at 24- and 48-h post-treatment for Western blot analysis. We also assessed the activity of the mTOR, AKT, ERK, and S6 signaling pathways. Our data analysis showed that at 24 and 48 h, the activities of the ERK and S6 pathways were significantly reduced by RMC-6236 treatment, increased by AUM-302 treatment, and notably inhibited by combinatorial treatment (Figures 6A,B). Notably, during the 48 h of treatment, we observed a significant reduction in the activity of three tested signaling pathways (mTOR, ERK, and S6) as measured by the phosphorylation status of their major components. Specifically, we noticed a reduction in phosphorylated mTOR levels between DMSO-treated hT1 and hT1 treated with combinations of 300 nM RMC-6236 and 100 nM AUM-302. A significant reduction in phosphorylated mTOR was observed between combinations of 300 nM RMC-6236 with 10nM and 100 nM AUM-302. Similar results were observed when the levels of phosphorylated ERK and S6 were assessed at 48 h post-treatment (Figure 6C).

In contrast, regulation of the ERK pathway is more complex. The levels of phosphorylated ERK 48 h after the treatment were significantly reduced by 300 nM RMC-6236 as compared to the DMSO-treated hT1 organoid; however, the 10nM and 100 nM of AUM-302 increased the levels of phosphorylated ERK (Figures 6A,B). Additionally, these levels were significantly reduced upon combinatorial treatment with RMC-6236 compared to the 10 nM and 100 nM of AUM-302 treatment. This suggests that with a longer duration of therapy, AUM-302 may cause a slight increase in ERK pathway activity, which is significantly diminished by RMC-6236. The levels of appropriate non-phosphorylated proteins were not significantly modified upon the treatment (Supplementary Figure 26).

In summary, our results demonstrate the potential synergy between PIM/AKT/mTOR and selective RAS inhibitors, providing an opportunity to test these therapeutic combinations in *in vivo* PDAC models and elucidate the mechanisms by which their combination leads to cancer cell growth inhibition and cell death.

## 4 Discussion

In the present study, we have demonstrated that AUM-302 effectively inhibits the growth of five tested PDAC spheroids compared to the DMSO-treated spheroids. Importantly, we showed that treating PDAC spheroids with GDC-0941 and TP-3654, PI3K and PIM inhibitors, respectively, at a wide range of concentrations was ineffective (Figures 1, 2; Supplementary Figures 1-14). While BEZ-235, a dual PI3K/mTOR inhibitor, and AUM-302, a triple PIM/PI3K/mTOR inhibitor, acted similarly with decreased growth, AUM-302 showed higher efficacy (Figures 1, 2; Supplementary Figures 1-14). Notably, AUM-302, in the range between 10e-6 and 10e-4, was able to reduce the growth of spheroids to 0%–20%, while the viability after treatment with BEZ oscillated between 25% and 75% within the same range of treatment (Figure 2).

Strikingly, the strong inhibitor effects of BEZ-235 and AUM-302 were maintained in PDAC organoids. Our results demonstrated that 100 nM and 1 μM BEZ-235 were able to reduce the viability of PDAC organoids by 25%–50% and 50%–75%, while AUM-302 was able to reduce the viability by 25%–50% and 75%–90%, respectively. These results showed that both tested compounds reduced PDAC organoid growth, with AUM-302 exhibiting significantly stronger effects (Figures 3, 4). In contrast, but similarly to the PDAC spheroids, GDC-0941 and TP-3654 did not show promising inhibitory effects (Figure 3; Supplementary Figures 15, 16). Thus, our subsequent analysis focused on the AUM-302s ability to reduce the growth of PDAC organoids as a single agent or in combination with the standard-of-care chemotherapy (gemcitabine) or the clinical trials RAS inhibitor, RMC-6236.

To better understand the consequences of AUM-302 on signaling pathway activity in PDAC organoids, we assessed pathway activity using Western blot analysis. The short-term treatment (24 h) with 10 nM of AUM-302 did not affected tested pathways (Supplementary Figure 17). However, we observed a trend toward modification of the activity of the mTOR, AKT, and S6 pathways upon treatment with 100 nM AUM-302, while others remained unaffected compared to the DMSO-treated organoids (Supplementary Figure 17). This observation aligns with previous data from PDAC cell line treatments (Ingle et al., 2023). The thorough analysis, with an expanded timeline (to 48 h) and treatment conditions (10 nM, 100 nM, and 1 μM), demonstrated that 1 μM of AUM302 has deleterious effects on the protein expression of most tested PDAC organoids, as it caused a significant reduction in their viability. Lower concentrations of AUM-302, to varying degrees, affected the expression of components of the PIM/PI3K/AKT pathway and its activation. This could reflect the mutational pattern of tested PDAC organoids and their heterogeneity. Thus, the combinatorial treatment assessed in this study could benefit the treatment.

The combinatorial approaches to treating PDAC, such as gemcitabine with nab-paclitaxel or FOLFIRINOX, to mention a few, have been shown to augment the efficacy of tested drugs (Neoptolemos et al., 2017; Miller et al., 2020; Sarantis et al., 2021; Lee et al., 2022; Koh et al., 2023). In this study, we evaluated whether AUM-302 can synergize with gemcitabine or RMC-6236 to inhibit PDAC organoid growth. Our data showed that a combination of AUM-302 and gemcitabine did not augment the efficacy of the compounds under tested conditions (Supplementary Figures 23, 24). Interestingly, AUM-302 synergized with RMC-6236 when tested in PDAC organoids (Figure 5; Supplementary Figure 25) at various concentrations of the drugs. Significantly, we showed that the combined treatment of 0.33 μM RMC-6236 and 0.1 μM AUM-302 resulted in almost 100% cell death in both hT1 and hM1a PDAC organoids. In addition, we assessed the impact of the combinatorial treatment versus a single agent on the hT1 PDAC organoid (Figure 6). After 24 h of treatment, we observed significant decreases in phospho-ERK1/2 and phospho-S6 activity, whereas phospho-mTOR and phospho-AKT were not significantly affected by single or combined RMC-6236 and AUM-302 treatment.

However, the 48-h treatment showed a consistent reduction in phospho-mTOR, phospho-ERK1/2, and phospho-S6 levels upon single RMC-6236 and combined RMC-6236 and AUM-302 treatment. Based on the limited data, RMC-6236 drives reductions in ERK and S6 activity. It is essential to note that we observed a transient increase in the activity of some of the tested pathways, which reflects a potential compensatory mechanism upon compound treatment. Furthermore, it has been shown that activation of the pro-survival pathway upon PIM inhibitor treatment is one of the mechanisms that may lead to acquired resistance (Li et al., 2020; Stulpinas et al., 2022). The varying responses to the PIM inhibitors could reflect the heterogeneity of the tested organoids and potentially the activation of compensatory mechanisms. It is worth noting that previous studies have shown that inhibition of PIM kinases can sensitize tumor cells to chemotherapeutic drugs, reduce growth, and increase apoptosis (Xu et al., 2011; Xu et al., 2016).

Further studies that comprehensively analyze transcriptomic and protein profiles of combination versus single-agent treatment should provide a better understanding of the impact of the tested compounds. In summary, we demonstrated that AUM-302, a triple PIM/PI3K/mTOR inhibitor, exhibits significant efficacy in inhibiting the viability of spheroids and organoids and synergizes with this inhibition to augment its effectiveness. This promising feature of the AUM-302 compound may offer potential therapeutic advantages in PDAC treatment, especially when combined with RAS and selective KRAS inhibitors.

## Data Availability

The original contributions presented in the study are included in the article/Supplementary Material, further inquiries can be directed to the corresponding author.

## Supplementary material

SUPPLEMENTARY FIGURE 1 PIM/PI3K/mTOR inhibitors decrease the growth of BxPC-3 cells 24 h post-treatment. **(A)** BEZ-235, **(B)** GDC-0941, **(C)** TP-3654, and **(D)** AUM-302. BxPC-3 PDAC cell line was treated as spheroids with DMSO (vehicle) and 1 nM, 10n M, 50 nM, 100 nM, and 1 μM concentrations of BEZ-235, GDC-0941, TP-3654, or AUM-302 for 24 h. The figure displays representative images from a sample of *N = 5*. The scale bar depicts 100 μm.

SUPPLEMENTARY FIGURE 2 PIM/PI3K/mTOR inhibitors decrease the growth of BxPC-3 cells 48 h post-treatment. **(A)** BEZ-235, **(B)** GDC-0941, **(C)** TP-3654, and **(D)** AUM-302. BxPC-3 PDAC cell line was treated as spheroids with DMSO (vehicle) and 1 nM, 10nM, 50 nM, 100 nM, and 1 μM concentrations of BEZ-235, GDC-0941, TP-3654, or AUM-302 for 48 h. The figure displays representative images from a sample of *N = 5*. The scale bar depicts 100 μm.

SUPPLEMENTARY FIGURE 3 PIM/PI3K/mTOR inhibitors decrease the growth of BxPC-3 cells 72 h post-treatment. **(A)** BEZ-235, **(B)** GDC-0941, **(C)** TP-3654, and **(D)** AUM-302. BxPC-3 PDAC cell line was treated as spheroids with DMSO (vehicle) and 1 nM, 10nM, 50 nM, 100 nM, and 1 μM concentrations of BEZ-235, GDC-0941, TP-3654, or AUM-302 for 72 h. The figure displays representative images from a sample of *N = 5*. The scale bar depicts 100 μm.

SUPPLEMENTARY FIGURE 4 PIM/PI3K/mTOR inhibitors decrease the growth of MIA PaCa-2 cells 24 h post-treatment. **(A)** BEZ-235, **(B)** GDC-0941, **(C)** TP-3654, and **(D)** AUM-302. MIA PaCa-2 PDAC cell line was treated as spheroids with DMSO (vehicle) and 1 nM, 10 nM, 50 nM, 100 nM, and 1 μM concentrations of BEZ-235, GDC-0941, TP-3654, or AUM-302 for 24 h. The figure displays representative images from a sample of *N = 5*. The scale bar depicts 100 μm.

SUPPLEMENTARY FIGURE 5 PIM/PI3K/mTOR inhibitors decrease the growth of MIA PaCa-2 cells 48 h post-treatment. **(A)** BEZ-235, **(B)** GDC-0941, **(C)** TP-3654, and **(D)** AUM-302. MIA PaCa-2 PDAC cell line was treated as spheroids with DMSO (vehicle) and 1 nM, 10 nM, 50 nM, 100 nM, and 1 μM concentrations of BEZ-235, GDC-0941, TP-3654, or AUM-302 for 48 h. The figure displays representative images from a sample of *N = 5*. The scale bar depicts 100 μm.

SUPPLEMENTARY FIGURE 6 PIM/PI3K/mTOR inhibitors decrease the growth of MIA PaCa-2 cells 72 h post-treatment. **(A)** BEZ-235, **(B)** GDC-0941, **(C)** TP-3654, and **(D)** AUM-302. MIA PaCa-2 PDAC cell line was treated as spheroids with DMSO (vehicle) and 1 nM, 10nM, 50 nM, 100 nM, and 1 μM concentrations of BEZ-235, GDC-0941, TP-3654, or AUM-302 for 72 h. The figure displays representative images from a sample of *N = 5*. The scale bar depicts 100 μm.

SUPPLEMENTARY FIGURE 7 PIM/PI3K/mTOR inhibitors decrease the growth of PANC-1 cells 24 h post-treatment. **(A)** BEZ-235, **(B)** GDC-0941, **(C**) TP-3654, and **(D)** AUM-302. PANC-1 PDAC cell line was treated as spheroids with DMSO (vehicle) and 1 nM, 10nM, 50 nM, 100 nM, and 1 μM concentrations of BEZ-235, GDC-0941, TP-3654, or AUM-302 for 24 h. The figure displays representative images from a sample of *N = 5*. The scale bar depicts 100 μm.

SUPPLEMENTARY FIGURE 8 PIM/PI3K/mTOR inhibitors decrease the growth of PANC-1 cells 48 h post-treatment. **(A)** BEZ-235, **(B)** GDC-0941, **(C)** TP-3654, and **(D)** AUM-302. PANC-1 PDAC cell line was treated as spheroids with DMSO (vehicle) and 1 nM, 10nM, 50 nM, 100 nM, and 1 μM concentrations of BEZ-235, GDC-0941, TP-3654, or AUM-302 for 48 h. The figure displays representative images from a sample of *N = 5*. The scale bar depicts 100 μm.

SUPPLEMENTARY FIGURE 9 PIM/PI3K/mTOR inhibitors decrease the growth of PANC-1 cells 72 h post-treatment. **(A)** BEZ-235, **(B)** GDC-0941, **(C)** TP-3654, and **(D)** AUM-302. PANC-1 PDAC cell line was treated as spheroids with DMSO (vehicle) and 1 nM, 10 nM, 50 nM, 100 nM, and 1 μM concentrations of BEZ-235, GDC-0941, TP-3654, or AUM-302 for 72 h. The figure displays representative images from a sample of *N = 5*. The scale bar depicts 100 μm.

SUPPLEMENTARY FIGURE 10 AUM-302 exerts a more substantial inhibitory growth effect in hF37 2D PDAC spheroids than other tested compounds. hF37 2D PDAC cell line was treated as spheroids with DMSO (vehicle) and various concentrations (1 nM - 100 μM) of BEZ-235 **(A)** GDC-0941 **(B)** TP-3654 **(C)** or AUM-302 **(D)** for 72 h. The figure displays representative images from *N = 5*. The scale bar depicts 100 μm.

SUPPLEMENTARY FIGURE 11 AUM-302 exerts a more substantial inhibitory growth effect in BxPC-3 PDAC spheroids than other tested compounds. BxPC-3 PDAC cell line was treated as spheroids with DMSO (vehicle) and various concentrations (1 nM - 100 μM) of BEZ-235 **(A)** GDC-0941 (**B**) TP-3654 **(C)** or AUM-302 **(D)** for 72 h. The figure displays representative images from *N = 5*. The scale bar depicts 100 μm.

SUPPLEMENTARY FIGURE 12 AUM-302 exerts a more substantial inhibitory growth effect in Capan-2 PDAC spheroids than other tested compounds. The Capan-2 PDAC cell line was treated as spheroids with DMSO (vehicle) and various concentrations (1 nM - 100 μM) of BEZ-235 **(A)** GDC-0941 **(B)** TP-3654 **(C)** or AUM-302 **(D)** for 72 h. The figure displays representative images from *N = 5*. The scale bar depicts 100 μm.

SUPPLEMENTARY FIGURE 13 AUM-302 exerts a more substantial inhibitory growth effect in MIA PaCa-2 PDAC spheroids than other tested compounds. MIA PaCa-2 PDAC cell line was treated as spheroids with DMSO (vehicle) and various concentrations (1 nM - 100 μM) of BEZ-235 **(A)** GDC-0941 **(B)** TP-3654 **(C)** or AUM-302 **(D)** for 72 h. The figure displays representative images from *a sample of N = 5*. The scale bar depicts 100 μm.

SUPPLEMENTARY FIGURE 14 AUM-302 exerts a more substantial inhibitory growth effect in PANC-1 PDAC spheroids than other tested compounds. PANC-1 PDAC cell line was treated as spheroids with DMSO (vehicle) and various concentrations (1 nM - 100 μM) of BEZ-235 **(A)** GDC-0941 **(B)** TP-3654 **(C)** or AUM-302 **(D)** for 72 h. The figure displays representative images from *N = 5*. The scale bar depicts 100 μm.

SUPPLEMENTARY FIGURE 15 AUM-302 exerts a more substantial inhibitory growth effect in hF31, hF37, hF44, hT1, and hM1a PDAC organoids than other tested compounds. hF31 **(A–D)** hF37 **(E–H)** hF44 **(I–L)** hT1 **(M–P)** and hM1a **(Q–T)** PDAC organoids were seeded in Matrigel and treated with DMSO (vehicle) and 10 nM, 100 nM, and 1 μM concentrations of GDC-0941 **(A,E,I,M,Q)** TP-3654 **(B,F,J,N,R)** BEZ-235 **(C,G,K,O,S)** or AUM-302 **(D,H,L,P,T)** for 72 h. The figure displays representative blow-up images from Figure 3. The scale bar depicts 100 μm.

SUPPLEMENTARY FIGURE 16 GDC-0941 and TP-3654 have weak inhibitory effects on PDAC organoids. hF31 **(A)** hF37 **(B)** hF44 **(C)** hT1 **(D)** and hM1a **(E)** PDAC organoids were seeded in Matrigel and treated with DMSO (vehicle) and 10nM, 100 nM, and 1 mM concentrations of GDC-0941 and TP-3654 for 72 h. At the end of the incubation, cell viability was measured using 3D Cell Titer-Glo. The results are shown as mean ± SD (*N = 5*) and normalized to the control (DMSO). The statistical analysis was performed using a two-way ANOVA in GraphPad Prism version 10.4.1. *p < 0.05, **p < 0.01, ***p < 0.001, and ****p < 0.0001.

SUPPLEMENTARY FIGURE 17 AUM-302 inhibits the activity of PI3K/mTOR/PIM pathways in PDAC organoids. hT1 and hM1a PDAC organoids were seeded in Matrigel and treated with DMSO (vehicle) and 10 nM and 100 nM AUM-302 for 24 h. Proteins were extracted as listed in the Materials and Methods sections.

SUPPLEMENTARY FIGURE 18 AUM-302s impact on the components of the PIM pathway in hF31 PDAC organoid after 24 and 48 h. hF31 PDAC organoid was seeded in Matrigel and treated with DMSO (vehicle) and 10nM, 100nM, and 1 μM AUM-302 for 24 **(A)** and 48 **(D)** hours. The results are shown as mean ± SD (*N = 5*) and normalized to the control (DMSO). Western blots were performed for mTOR, AKT, c-Myc, S6, phosphorylated counterparts, and β-Actin as a loading control. Densitometry analysis was conducted to quantify relative protein expression, normalized to β-actin control and non-phosphorylated proteins in samples collected 24 and 48 h post-treatment **(B,C,E,F)**. Densitometry was measured with ImageJ and analyzed with GraphPad Prism as described in “Materials and Methods.” Data are presented as mean ± SD values (*N* = 3). *, *P* < 0.05; **, *P* < 0.01; ***, *P* < 0.001; ****, *P* < 0.0001.

SUPPLEMENTARY FIGURE 19 AUM-302s impact on the components of the PIM pathway in hF37 PDAC organoid after 24 and 48 h. hF31 PDAC organoid was seeded in Matrigel and treated with DMSO (vehicle) and 10nM, 100nM, and 1 μM AUM-302 for 24 **(A)** and 48 **(D)** hours. The results are shown as mean ± SD (*N = 5*) and normalized to the control (DMSO). Western blots were performed for mTOR, AKT, c-Myc, S6, phosphorylated counterparts, and β-Actin as a loading control. Densitometry analysis was conducted to quantify relative protein expression, normalized to β-actin control and non-phosphorylated proteins in samples collected 24 and 48 h post-treatment **(B,C,E,F)**. Densitometry was measured with ImageJ and analyzed with GraphPad Prism as described in “Materials and Methods.” Data are presented as mean ± SD values (*N* = 3). *, *P* < 0.05; **, *P* < 0.01; ***, *P* < 0.001; ****, *P* < 0.0001.

SUPPLEMENTARY FIGURE 20 AUM-302s impact on the components of the PIM pathway in hF44 PDAC organoid after 24 and 48 h. hF31 PDAC organoid was seeded in Matrigel and treated with DMSO (vehicle) and 10nM, 100nM, and 1 μM AUM-302 for 24 **(A)** and 48 **(D)** hours. The results are shown as mean ± SD (*N = 5*) and normalized to the control (DMSO). Western blots were performed for mTOR, AKT, c-Myc, S6, phosphorylated counterparts, and β-Actin as a loading control. Densitometry analysis was conducted to quantify relative protein expression, normalized to β-actin control and non-phosphorylated proteins in samples collected 24 and 48 h post-treatment **(B,C,E,F)**. Densitometry was measured with ImageJ and analyzed with GraphPad Prism as described in “Materials and Methods.” Data are presented as mean ± SD values (*N* = 3). *, *P* < 0.05; **, *P* < 0.01; ***, *P* < 0.001; ****, *P* < 0.0001.

SUPPLEMENTARY FIGURE 21 AUM-302s impact on the components of the PIM pathway in hT1 PDAC organoid after 24 and 48 h. hF31 PDAC organoid was seeded in Matrigel and treated with DMSO (vehicle) and 10 nM, 100nM, and 1 μM AUM-302 for 24 **(A)** and 48 **(D)** hours. The results are shown as mean ± SD (*N = 5*) and normalized to the control (DMSO). Western blots were performed for mTOR, AKT, c-Myc, S6, phosphorylated counterparts, and β-Actin as a loading control. Densitometry analysis was conducted to quantify relative protein expression, normalized to β-actin control and non-phosphorylated proteins in samples collected 24 and 48 h post-treatment **(B,C,E,F)**. Densitometry was measured with ImageJ and analyzed with GraphPad Prism as described in “Materials and Methods.” Data are presented as mean ± SD values (*N* = 3). *, *P* < 0.05; **, *P* < 0.01; ***, *P* < 0.001.

SUPPLEMENTARY FIGURE 22 AUM-302s impact on the components of the PIM pathway in hM1a PDAC organoid after 24 and 48 h. hF31 PDAC organoid was seeded in Matrigel and treated with DMSO (vehicle) and 10 nM, 100 nM, and 1 μM AUM-302 for 24 **(A)** and 48 **(D)** hours. The results are shown as mean ± SD (*N = 5*) and normalized to the control (DMSO). Western blots were performed for mTOR, AKT, c-Myc, S6, phosphorylated counterparts, and β-Actin as a loading control. Densitometry analysis was conducted to quantify relative protein expression, normalized to β-actin control and non-phosphorylated proteins in samples collected 24 and 48 h post-treatment **(B,C,E,F)**. Densitometry was measured with ImageJ and analyzed with GraphPad Prism as described in “Materials and Methods.” Data are presented as mean ± SD values (*N* = 3). *, *P* < 0.05; **, *P* < 0.01; ***, *P* < 0.001; ****, *P* < 0.0001.

SUPPLEMENTARY FIGURE 23 AUM-302 shows a weak synergy with gemcitabine in the hT1 PDAC organoids. hT1 PDAC organoids were seeded in Matrigel and treated with various concentrations of AUM-302, Gemcitabine, or a combination thereof. Seventy-two hours post-treatment, the cell viability was assessed using 3D Cell Titer-Glo and analyzed using a non-linear regression model of log(inhibitor) vs. response–variable slope (four parameters) equation in GraphPad Prism version 10.4.1. and Synergy Finder. **(A)** Cell viability of mono-and combinatorial treatment. The results are shown as mean ± SD (*N = 5*) and normalized to the control (DMSO). **(B)** The HSA Synergy Score is a dose matrix inhibition response. **(C)** The HSA Synergy Score is shown as a plot contour. **(D)** The graph was generated using a Plot Barometer using viability results. The needle points to the observed growth inhibition, denoted in percentage values, for the synergy between AUM-302 (0.00023 μM) and gemcitabine (0.33 μM). The four white lines represent the predicted inhibition values calculated by the HSA, Loewe, ZIP, and BLISS models at specific concentrations.

SUPPLEMENTARY FIGURE 24 AUM-302 shows a weak synergy with gemcitabine in the hM1A PDAC organoids. hM1A PDAC organoids were seeded in Matrigel and treated with various concentrations of AUM-302, gemcitabine, and a combination thereof. Seventy-two hours post-treatment, the cell viability was assessed using 3D Cell Titer-Glo and analyzed using a non-linear regression model of log(inhibitor) vs. response–variable slope (four parameters) equation in GraphPad Prism version 10.4.1. and Synergy Finder. **(A)** Cell viability of mono-and combinatorial treatment. The results are shown as mean ± SD (*N = 5*) and normalized to the control (DMSO). **(B)** The HSA Synergy Score is a dose matrix inhibition response. **(C)** The HSA Synergy Score is shown as a plot contour. **(D)** The graph was generated using a Plot Barometer using viability results. The needle points to the observed growth inhibition, denoted in percentage value, for the synergy between AUM-302 (0.00066 μM) and gemcitabine (0.1 μM). The four white lines represent the predicted inhibition values calculated by the HSA, Loewe, ZIP, and BLISS models at specific concentrations.

SUPPLEMENTARY FIGURE 25 AUM-302 synergizes with RMC-6236 to inhibit the growth of hM1A PDAC organoids. hM1A PDAC organoids were seeded in Matrigel and treated with various concentrations of AUM-302, RMC-6236, and a combination of both compounds. Seventy-two hours post-treatment, the cell viability was assessed using 3D Cell Titer-Glo and analyzed using a non-linear regression model of log(inhibitor) vs. response–variable slope (four parameters) equation in GraphPad Prism version 10.4.1. and Synergy Finder. **(A)** Cell viability of mono-and combinatorial treatment. The results are shown as mean ± SD (*N = 5*) and normalized to the control (DMSO). **(B)** The HSA Synergy Score is a dose matrix inhibition response. **(C)** The HSA Synergy Score is shown as a plot contour. **(D)** The graph was generated using a Plot Barometer using viability results. The needle points to the observed growth inhibition, denoted in percentage values, for the synergy between AUM-302 0.1 μM and RMC-6236 0.33 μM. The four white lines represent the predicted inhibition values calculated by the HSA, Loewe, ZIP, and BLISS models at specific concentrations.

SUPPLEMENTARY FIGURE 26 AUM-302 and RMC-6236 impact on the total levels of components of tested signaling pathways in the hT1 PDAC organoids. hT1 organoids were treated with DMSO (vehicle), 300nM RMC-6236, 10 nM and 100 nM AUM-302, and combinations of thereof for 24 and 48 h **(A,B)** Densitometry analysis was conducted to quantify relative protein expression of total proteins normalized to β-actin control in samples after 24- and 48-h post-treatment. Densitometry was measured with ImageJ and analyzed with GraphPad Prism as described in “Materials and Methods.” Data are presented as mean ± SD values (*N* = 3).
