# Supplementary material for: AUM-302, a novel triple PIM/PI3K/mTOR inhibitor, synergizes with RAS inhibition and impedes the growth of pancreatic ductal adenocarcinoma spheroids and organoids
Source: Front Pharmacol. 2026 Feb 11;17:1685433. doi: 10.3389/fphar.2026.1685433 (PMC12932507; doi:10.3389/fphar.2026.1685433)
Supplement: Supplementary file 1 [file DataSheet1.PDF]

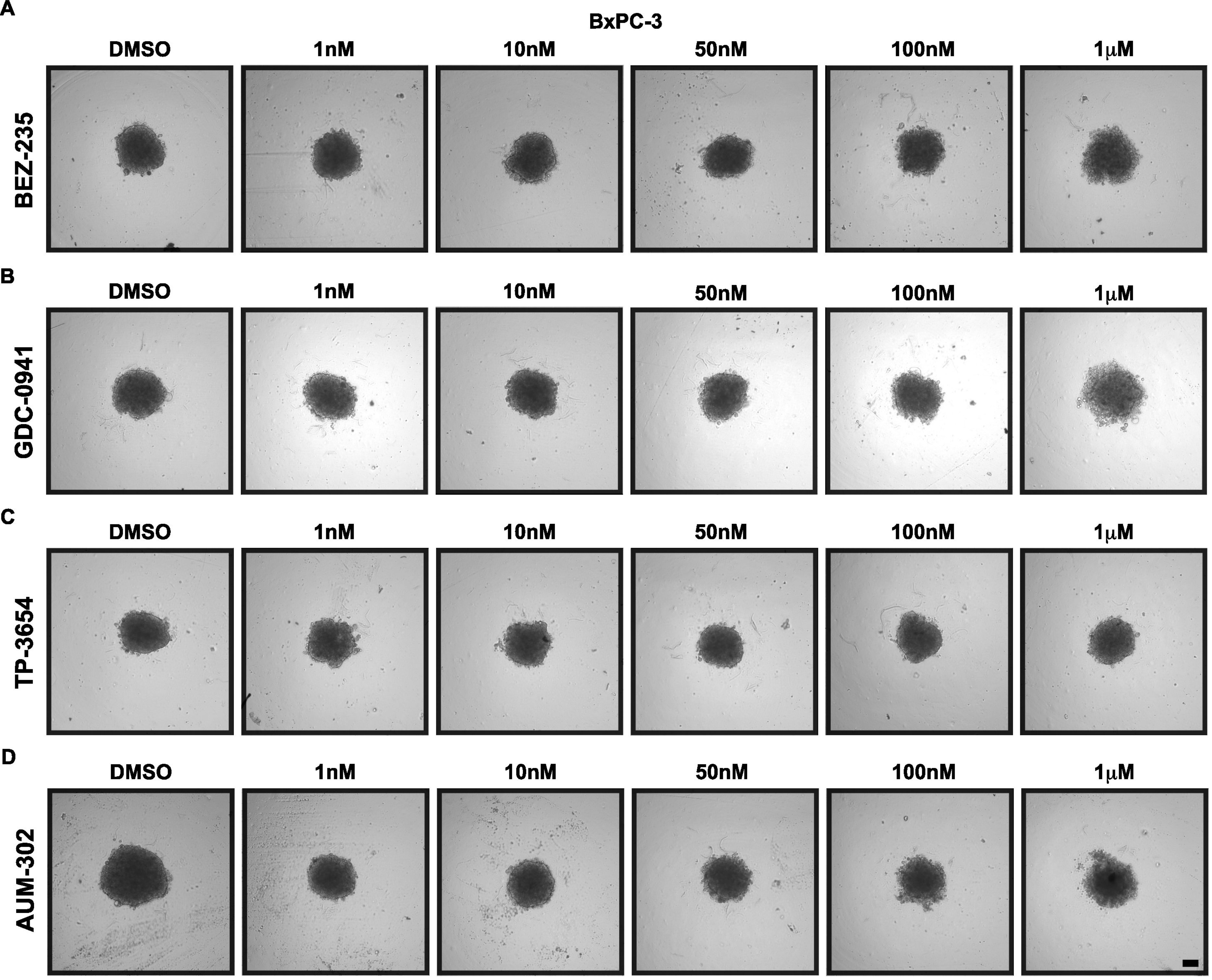

Supplementary Figure 1.

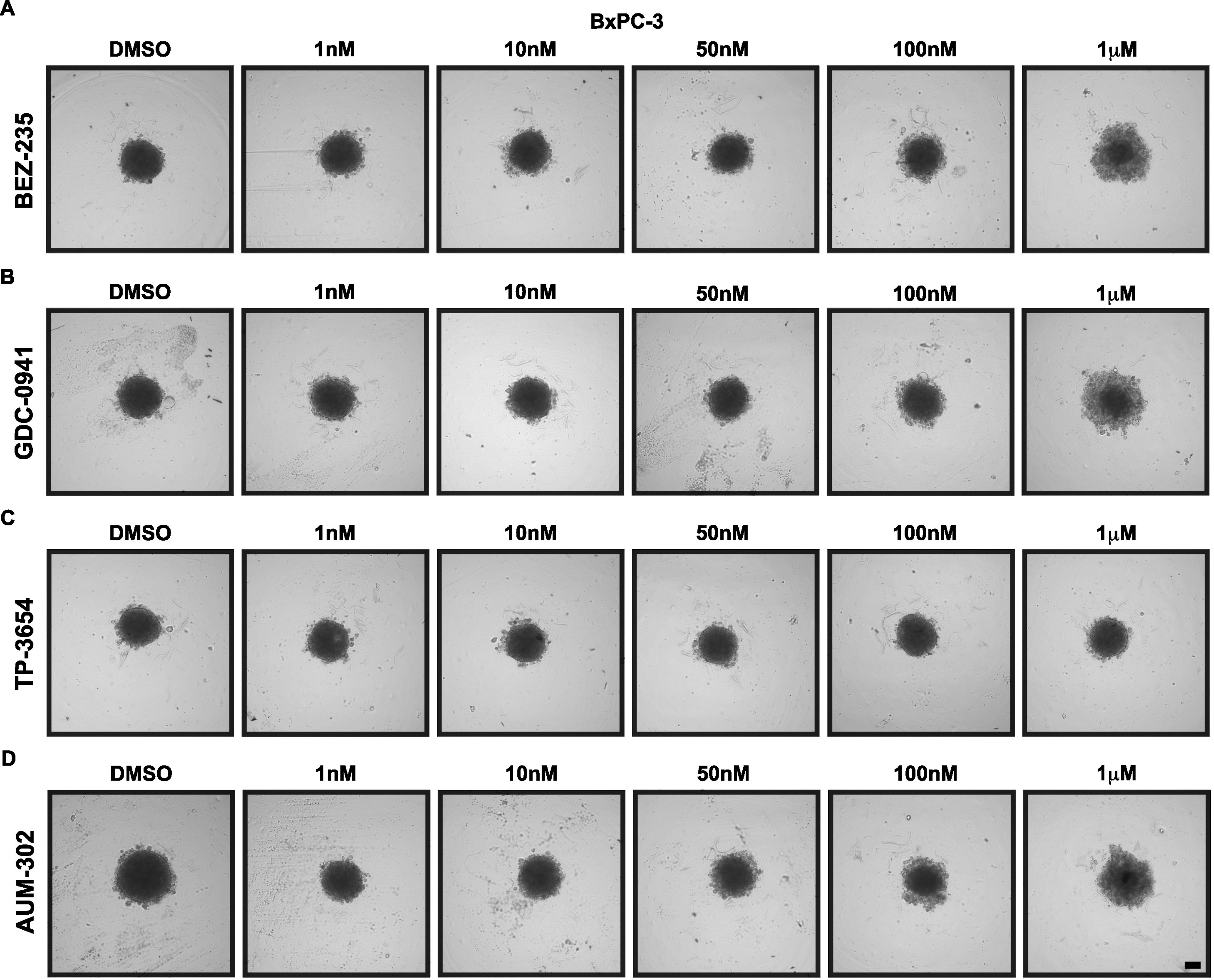

Supplementary Figure 2.

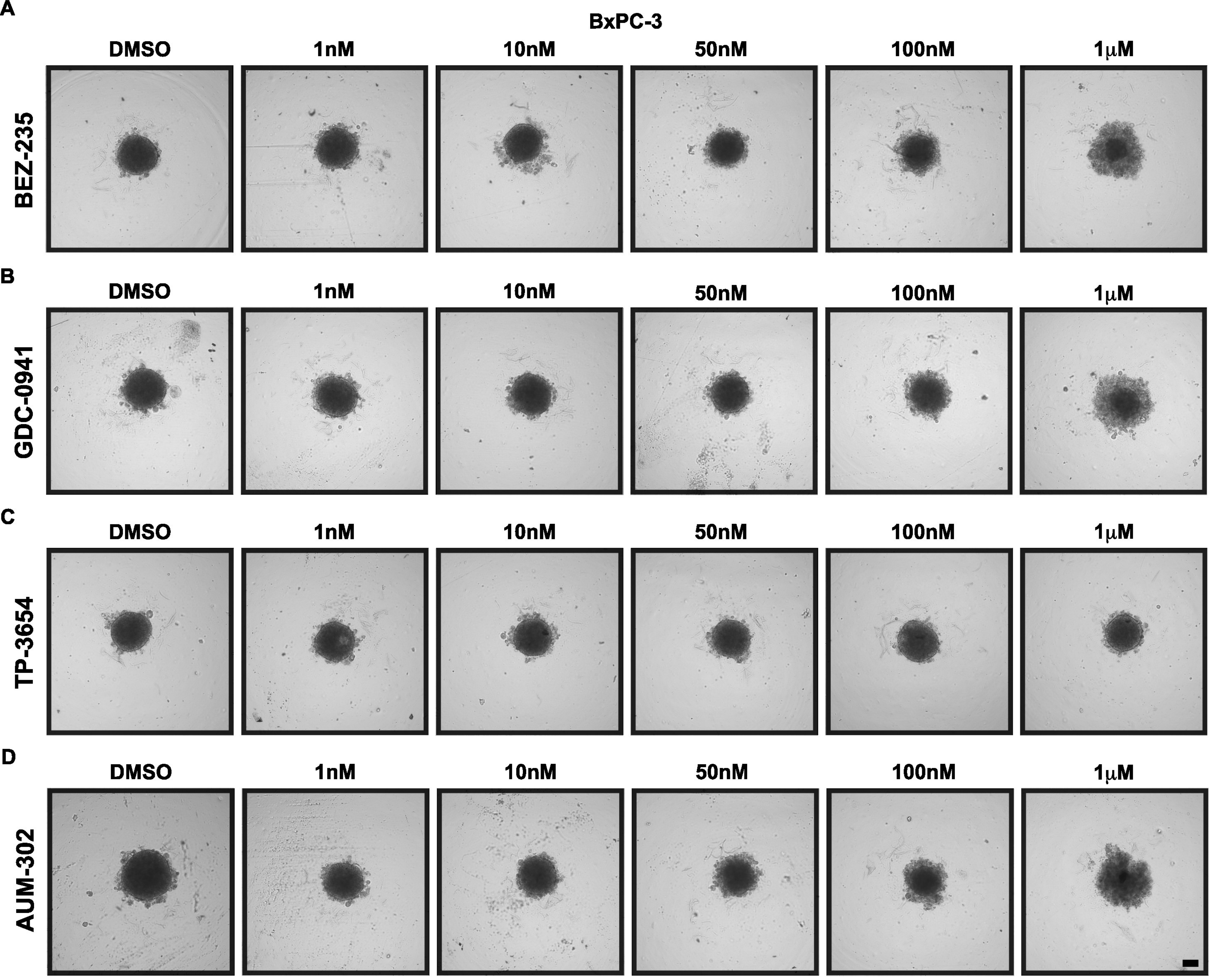

Supplementary Figure 3.

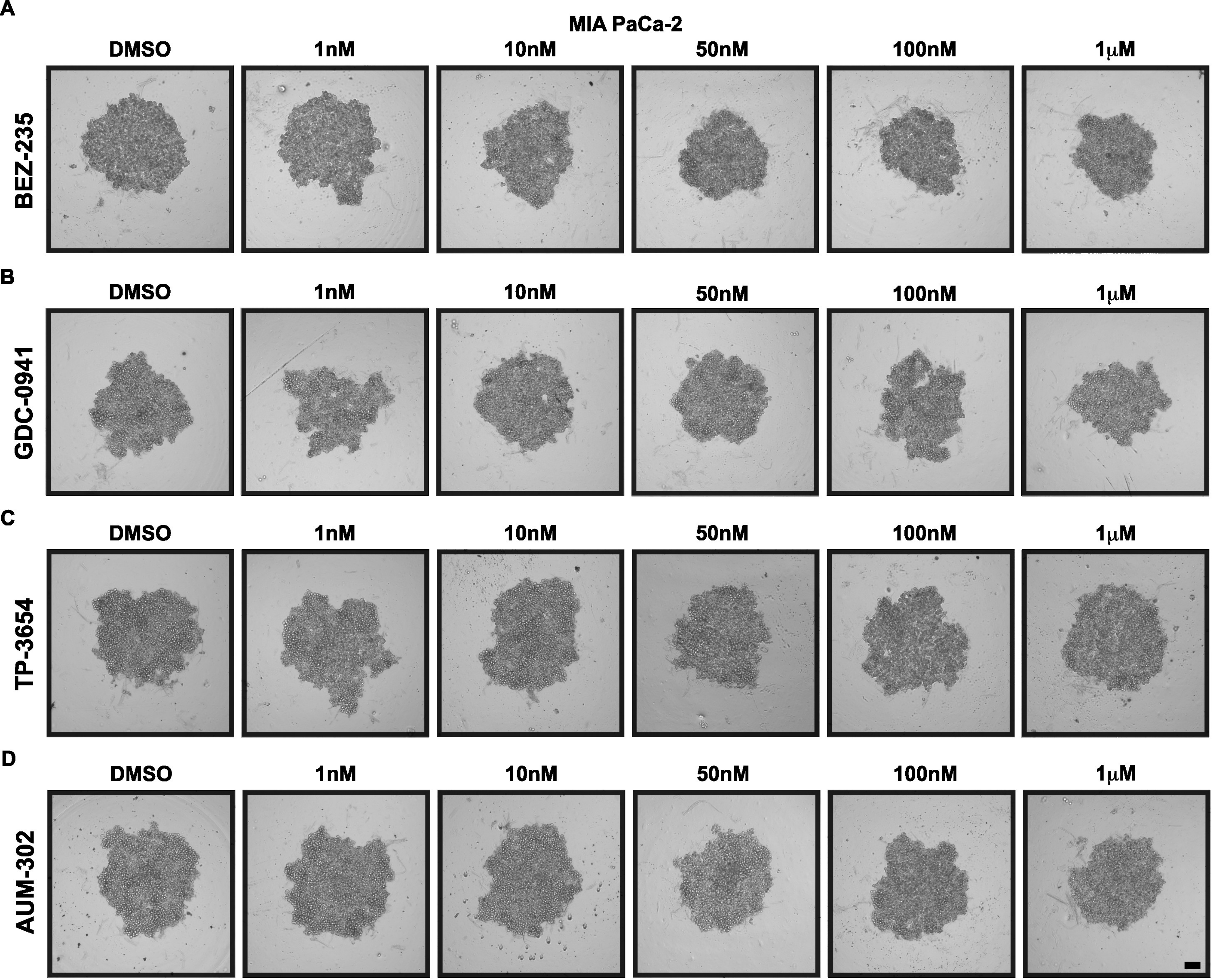

Supplementary Figure 4.

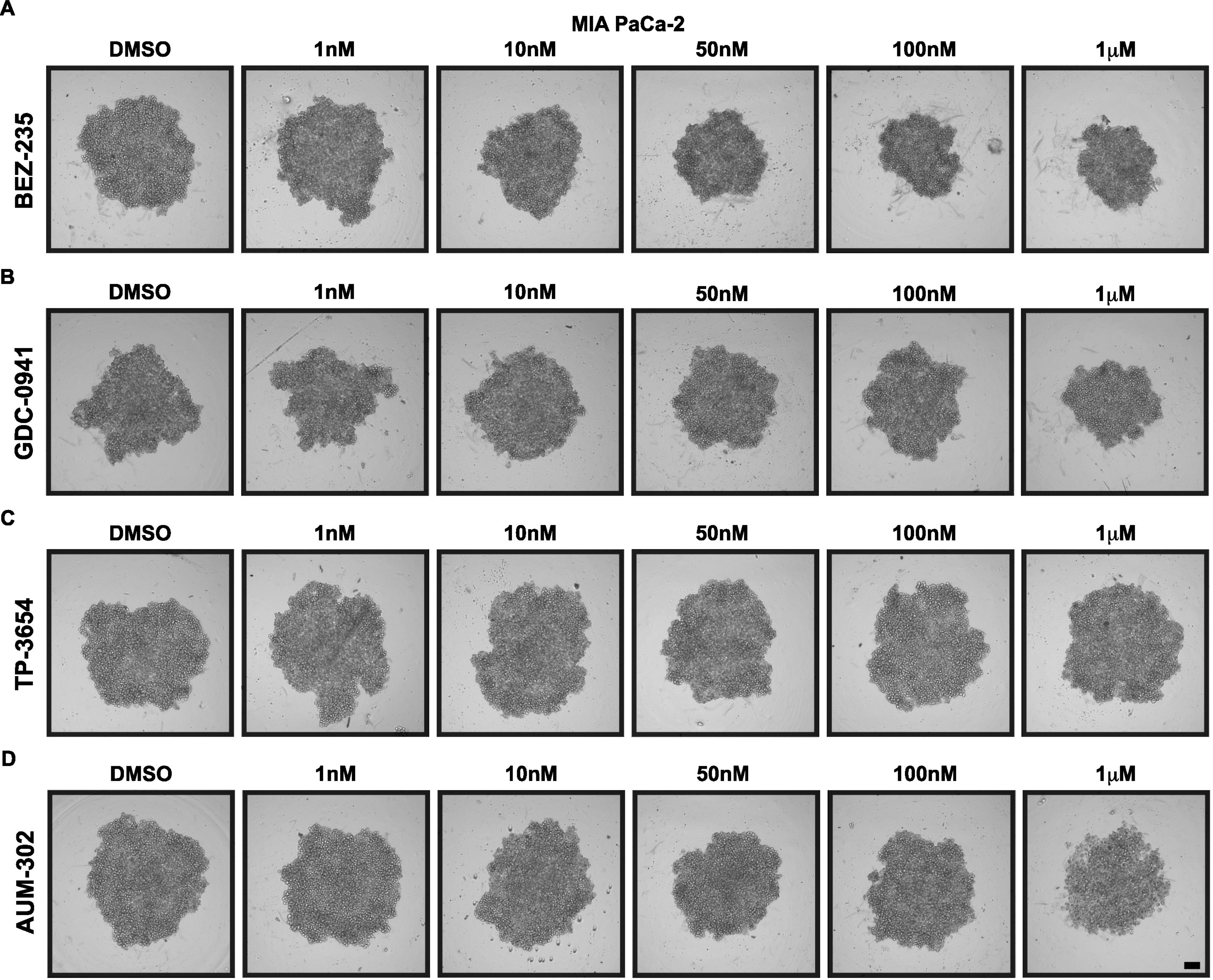

Supplementary Figure 5.

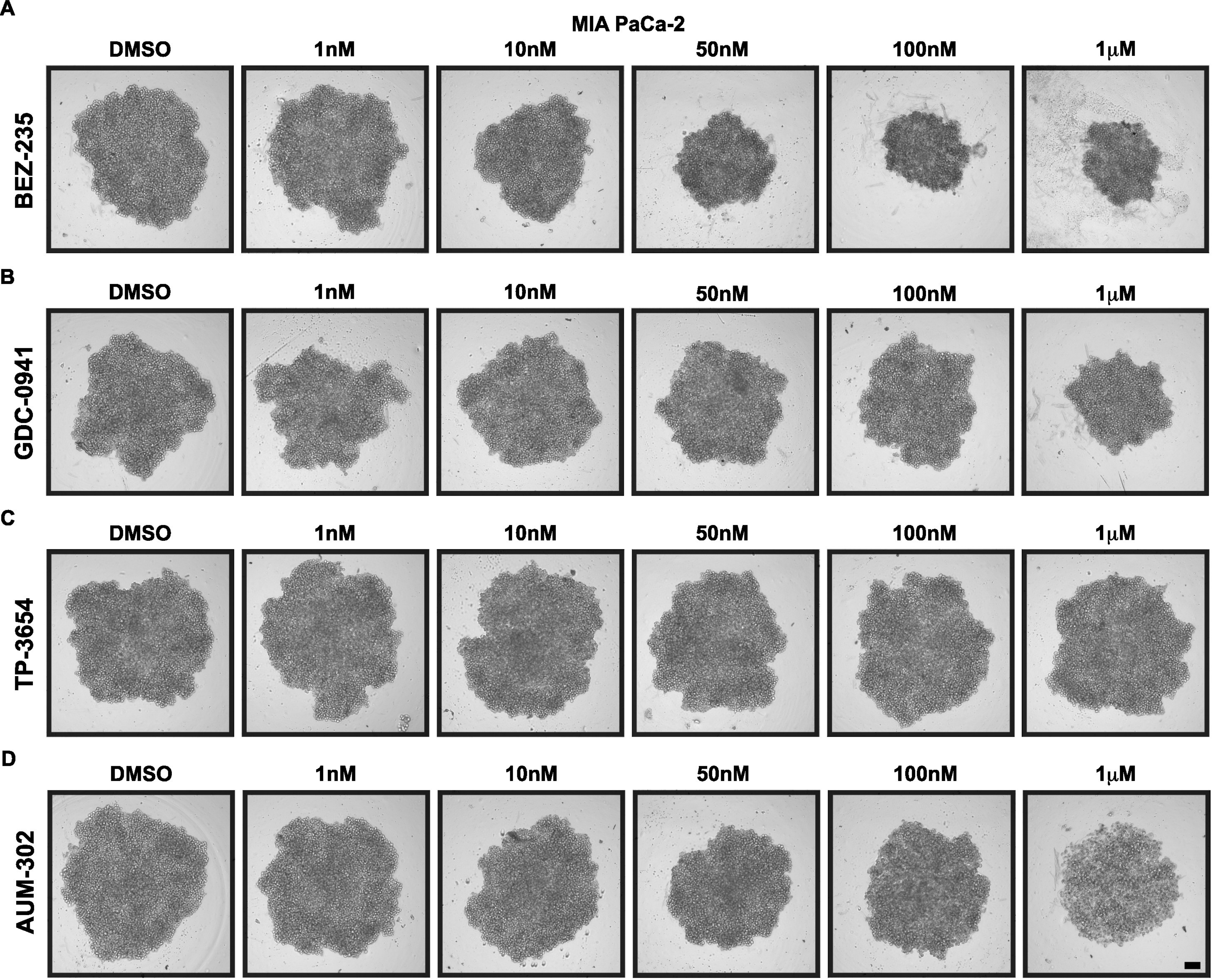

Supplementary Figure 6.

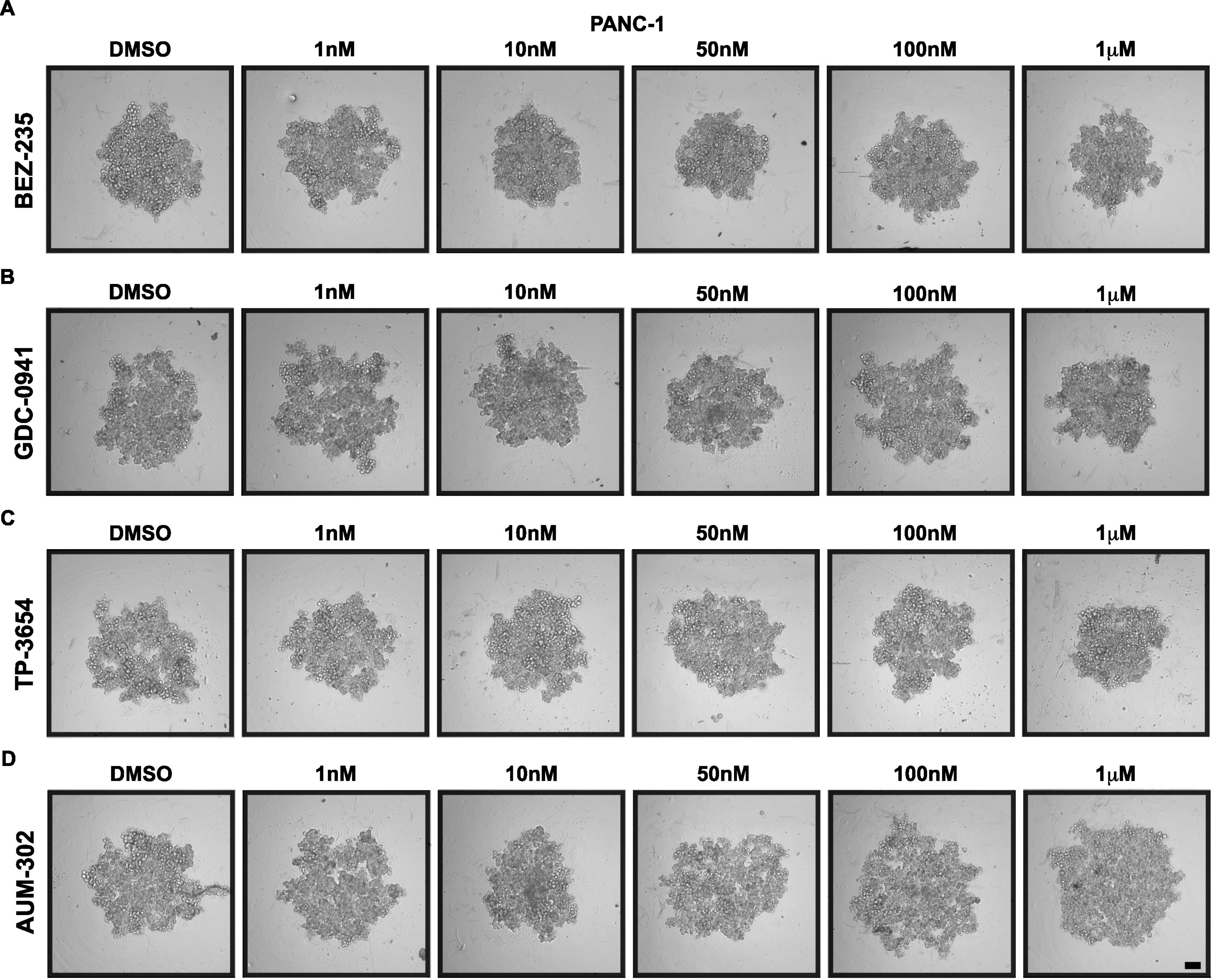

Supplementary Figure 7.

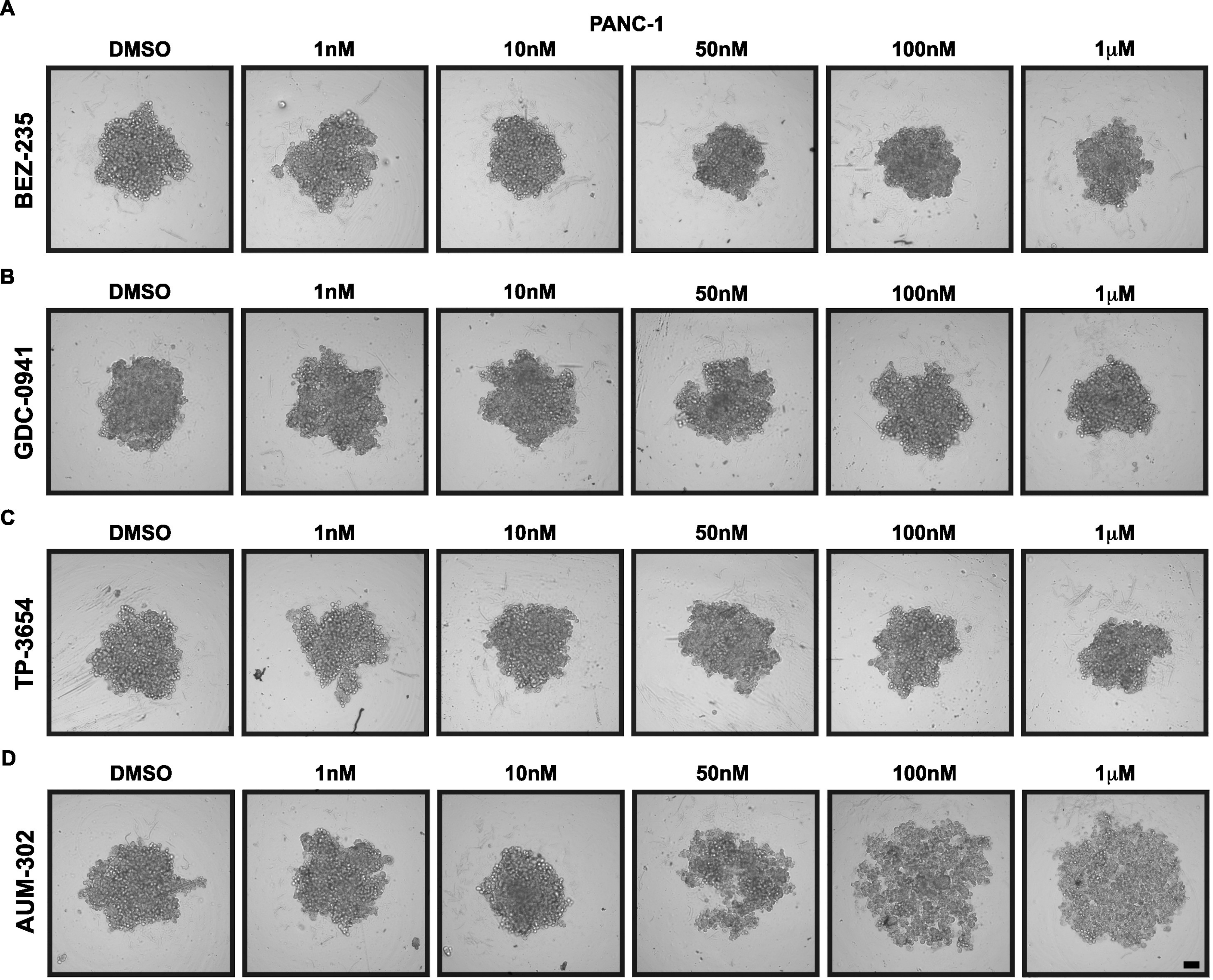

Supplementary Figure 8.

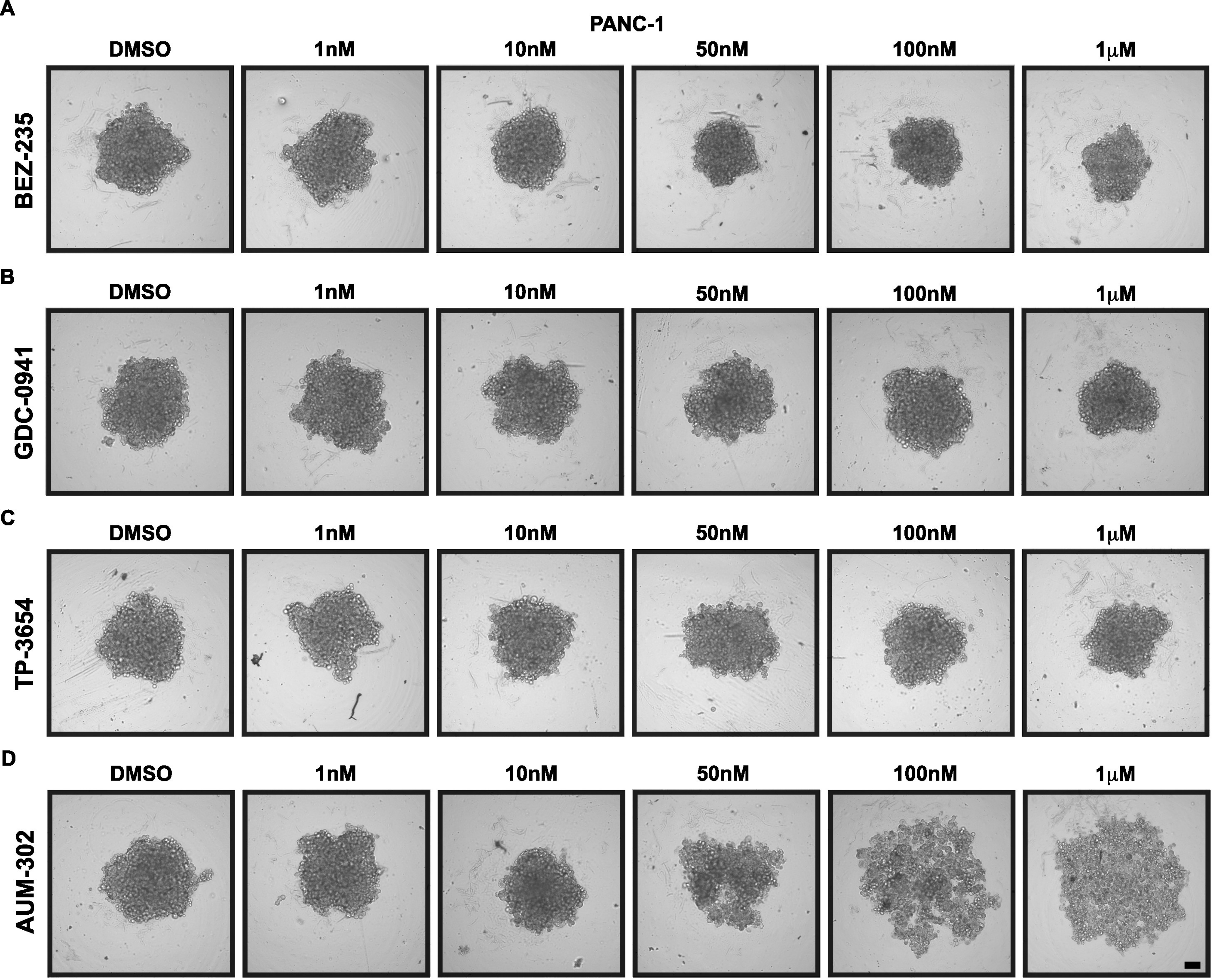

Supplementary Figure 9.

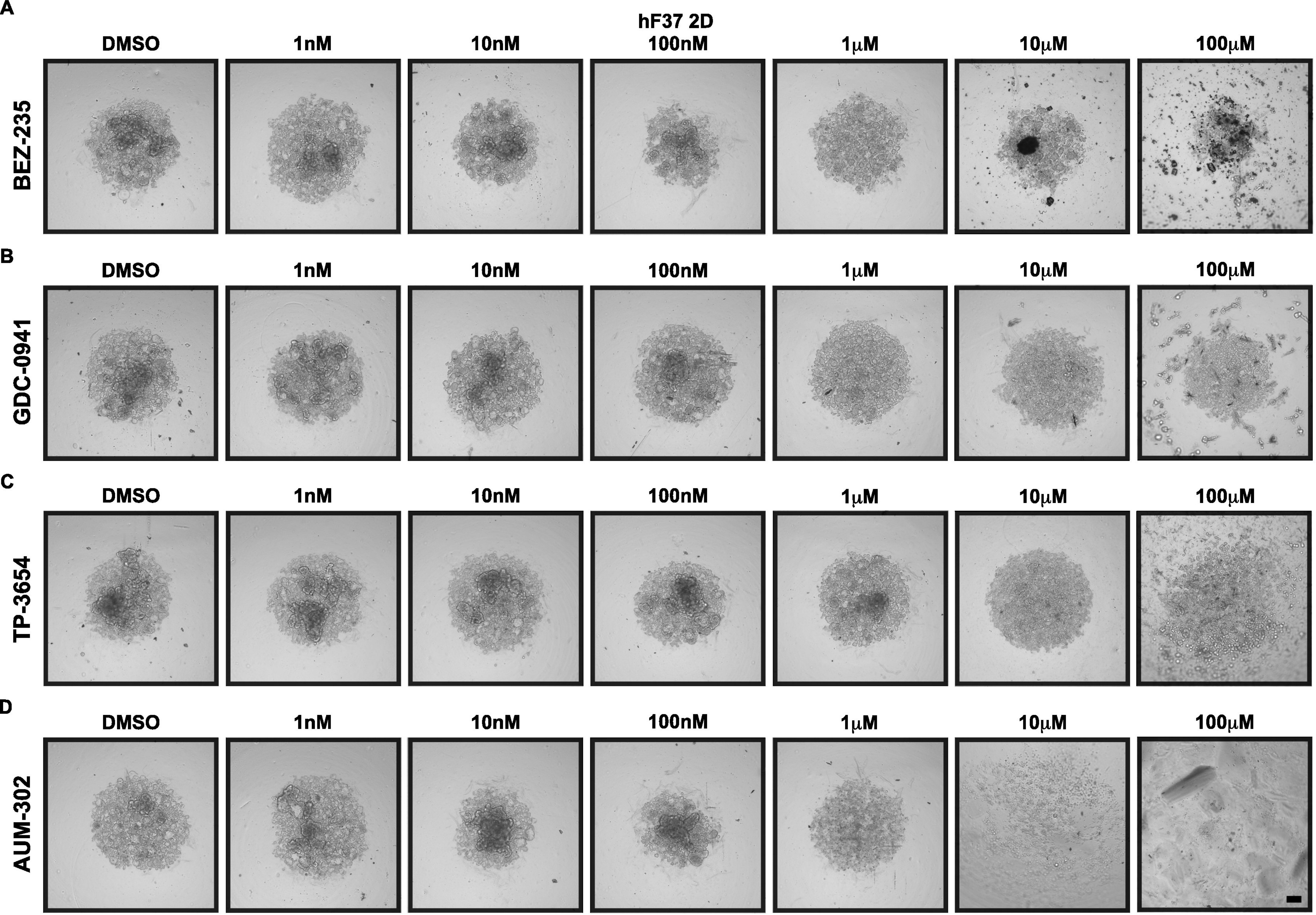

Supplementary Figure 10.

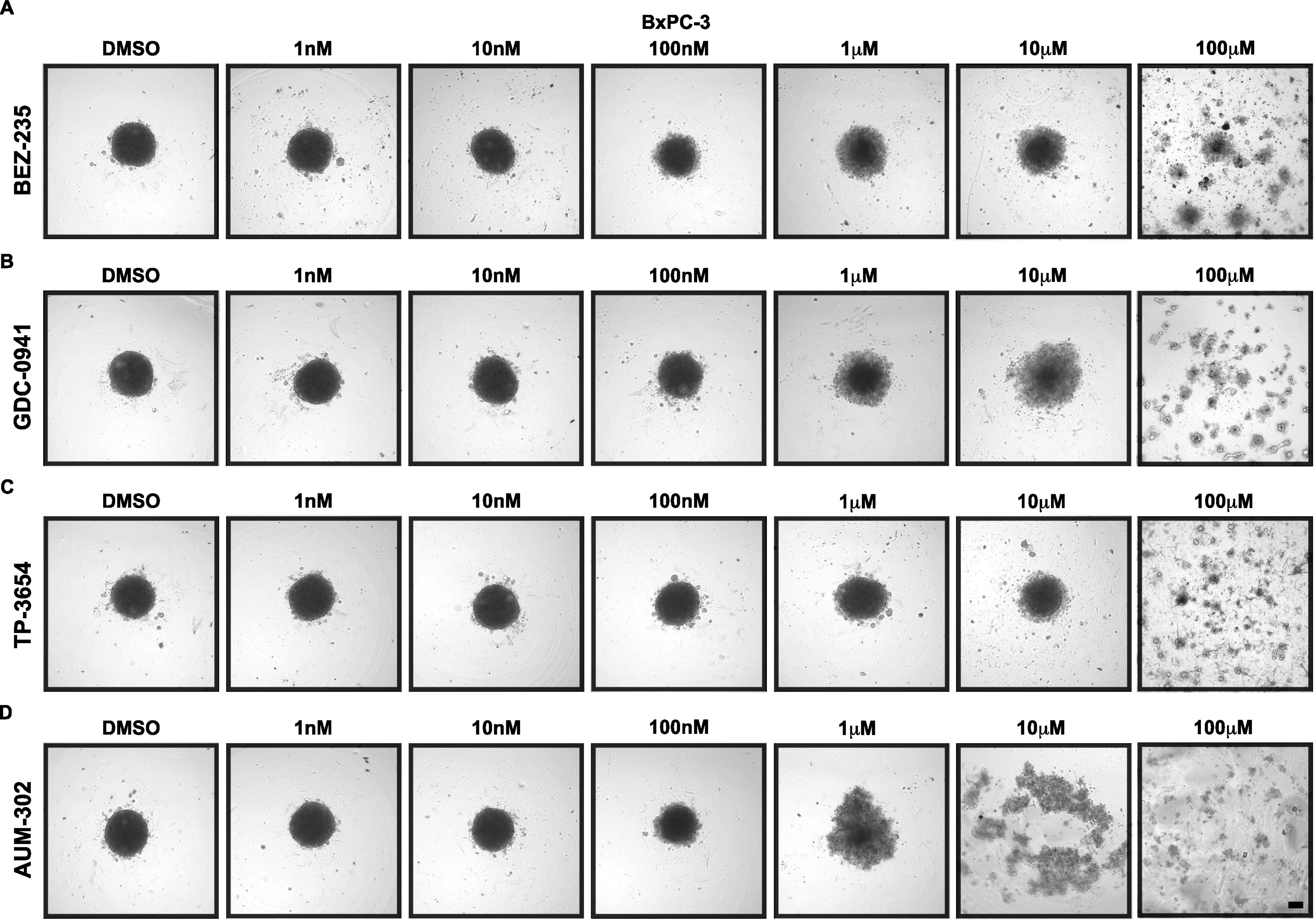

Supplementary Figure 11.

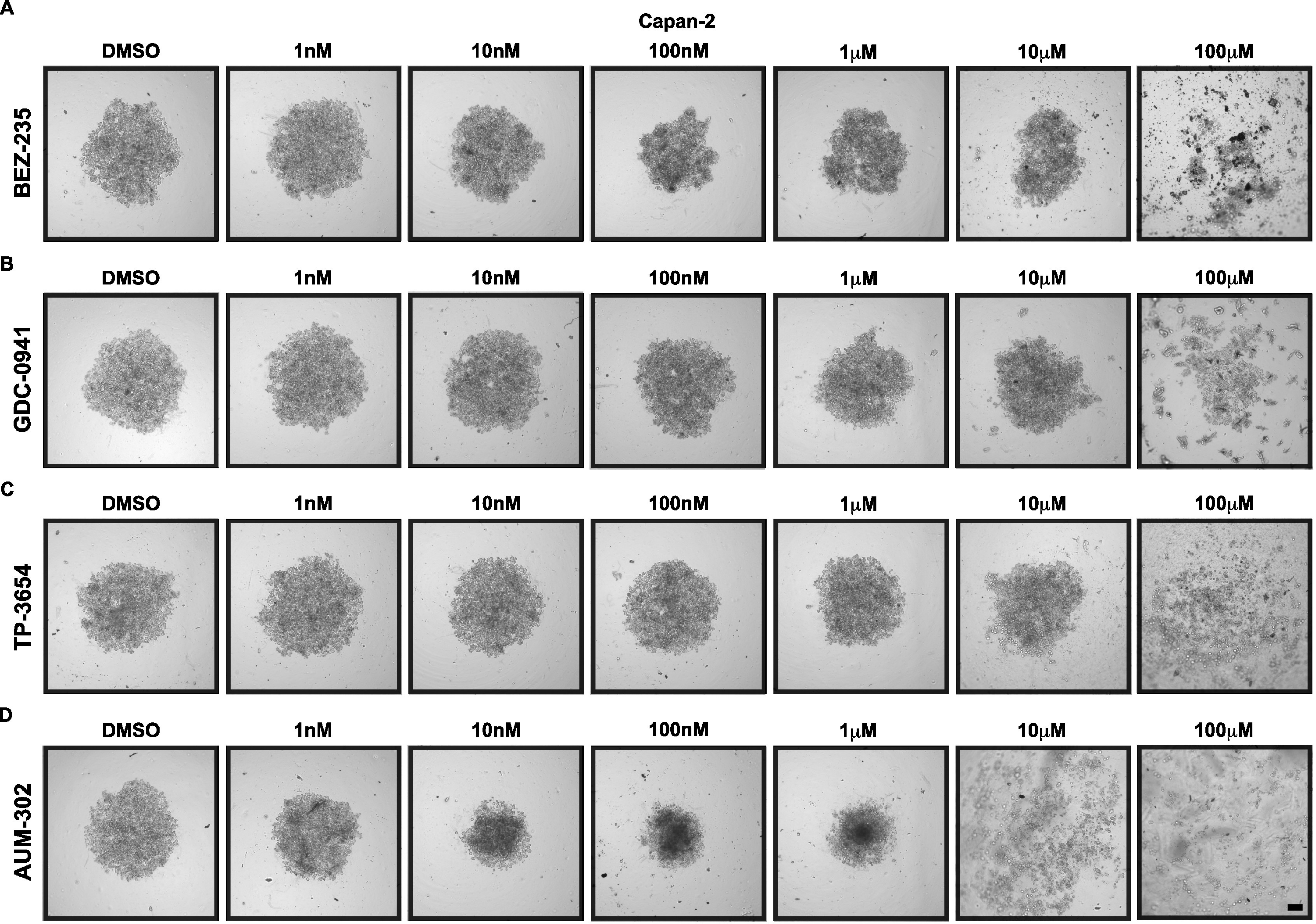

Supplementary Figure 12.

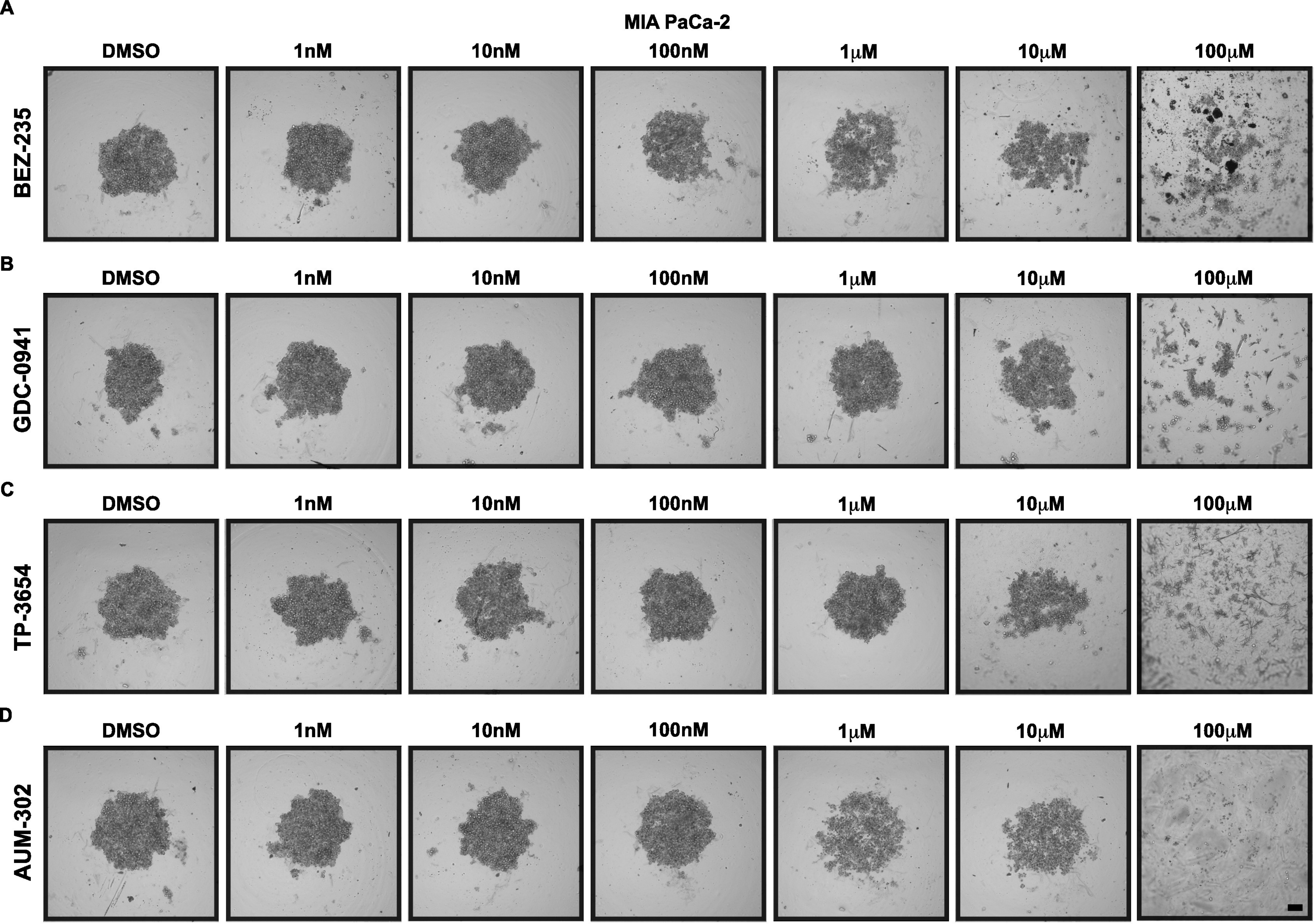

Supplementary Figure 13.

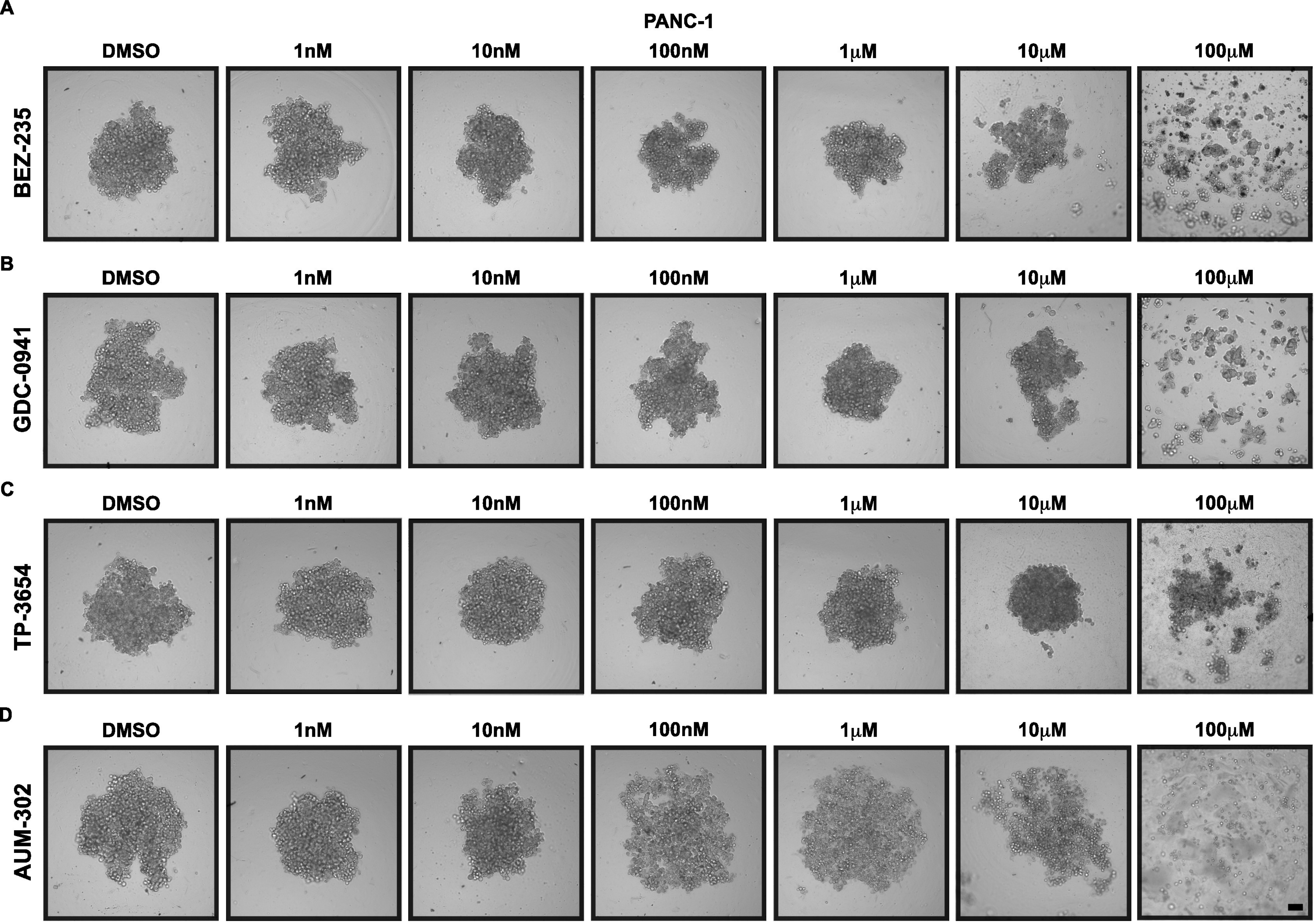

Supplementary Figure 14.

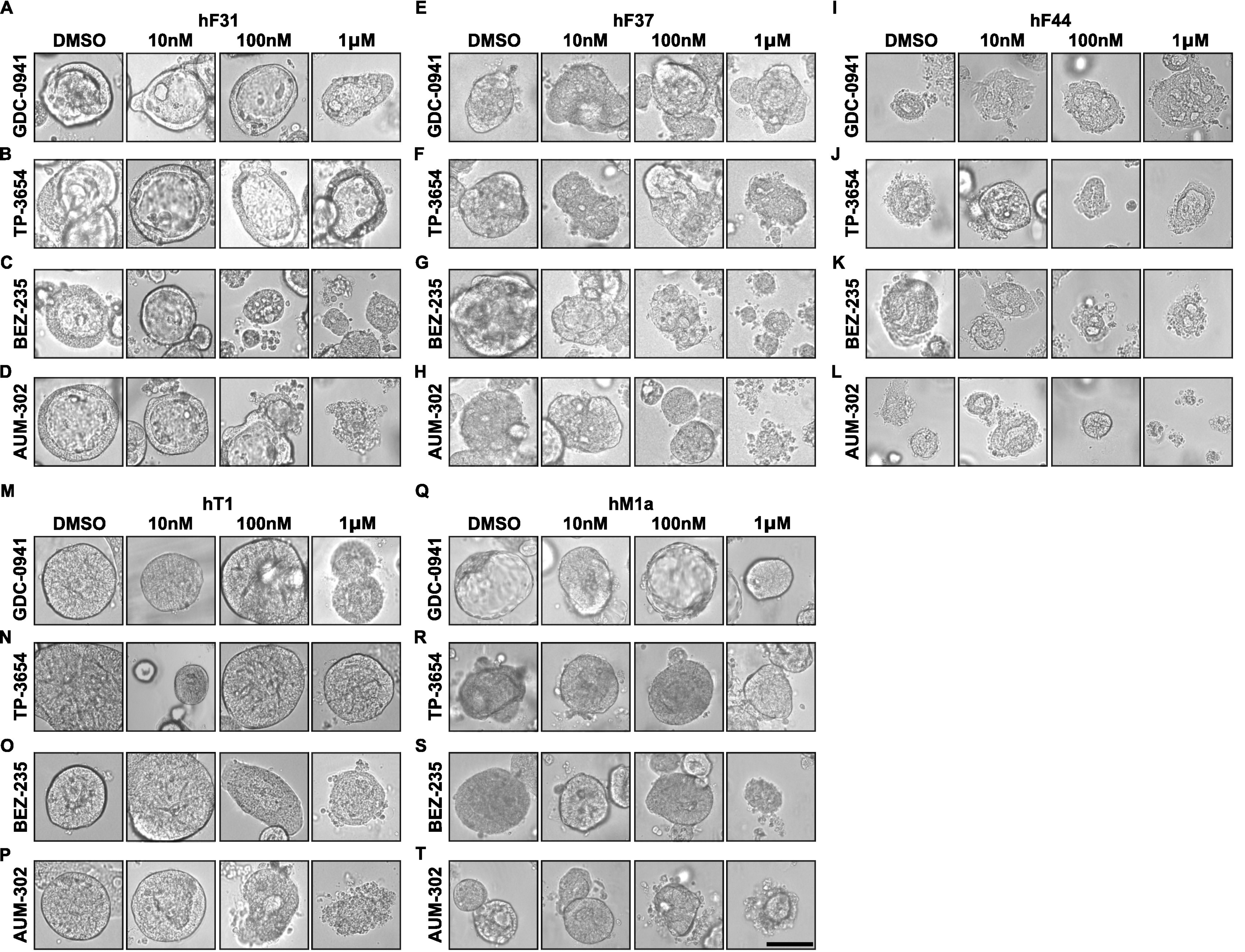

Supplementary Figure 15.

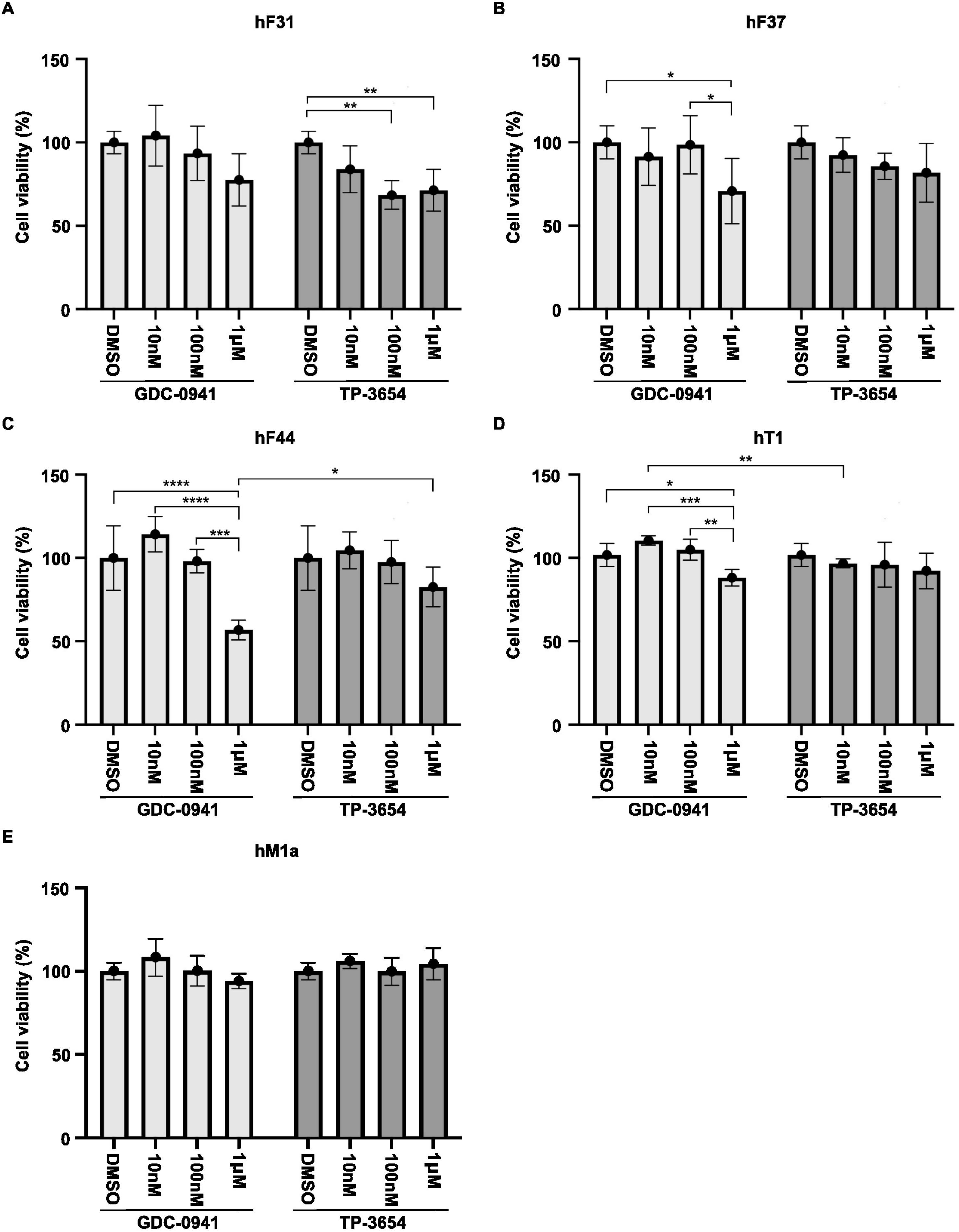

Supplementary Figure 16.

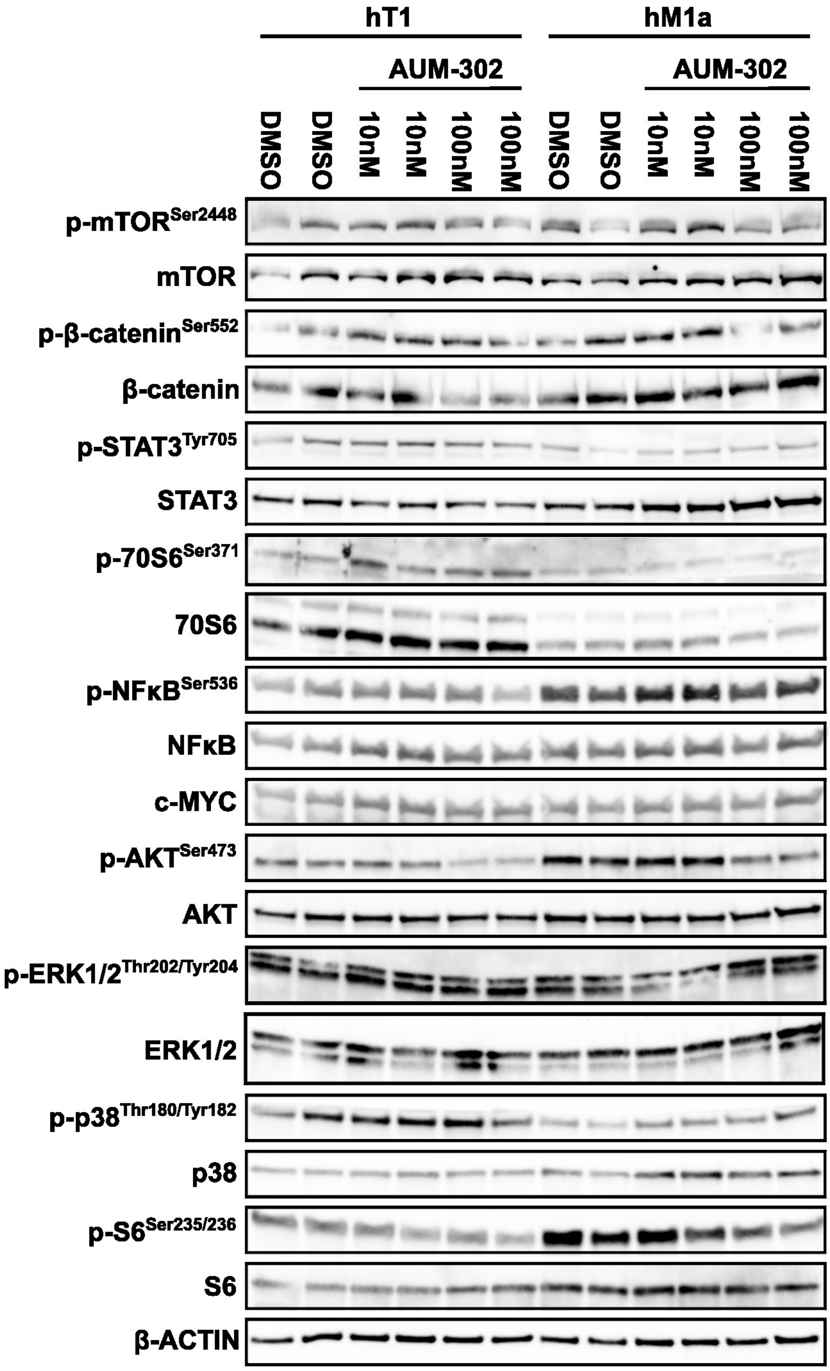

Supplementary Figure 17.

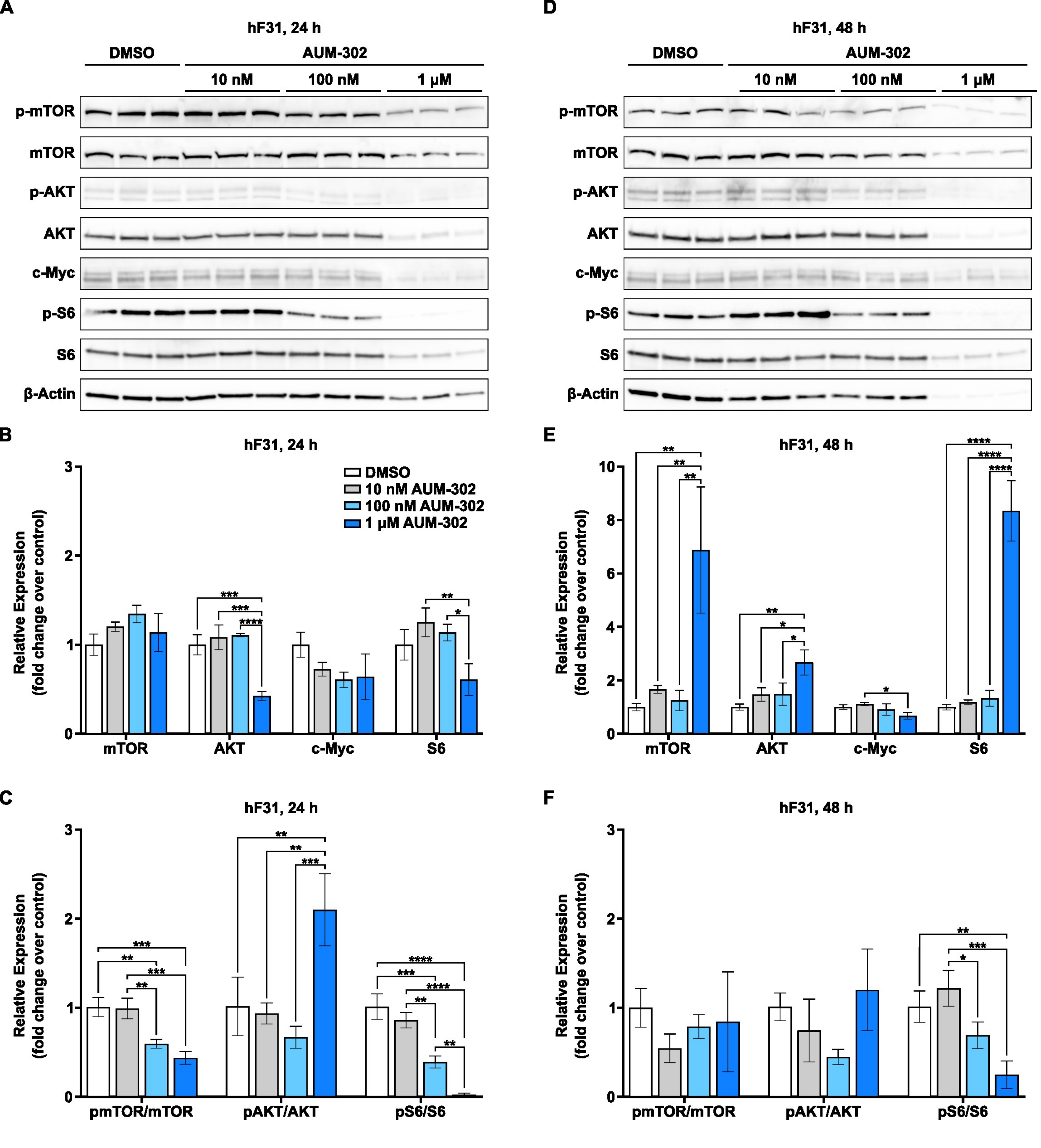

Supplementary Figure 18.

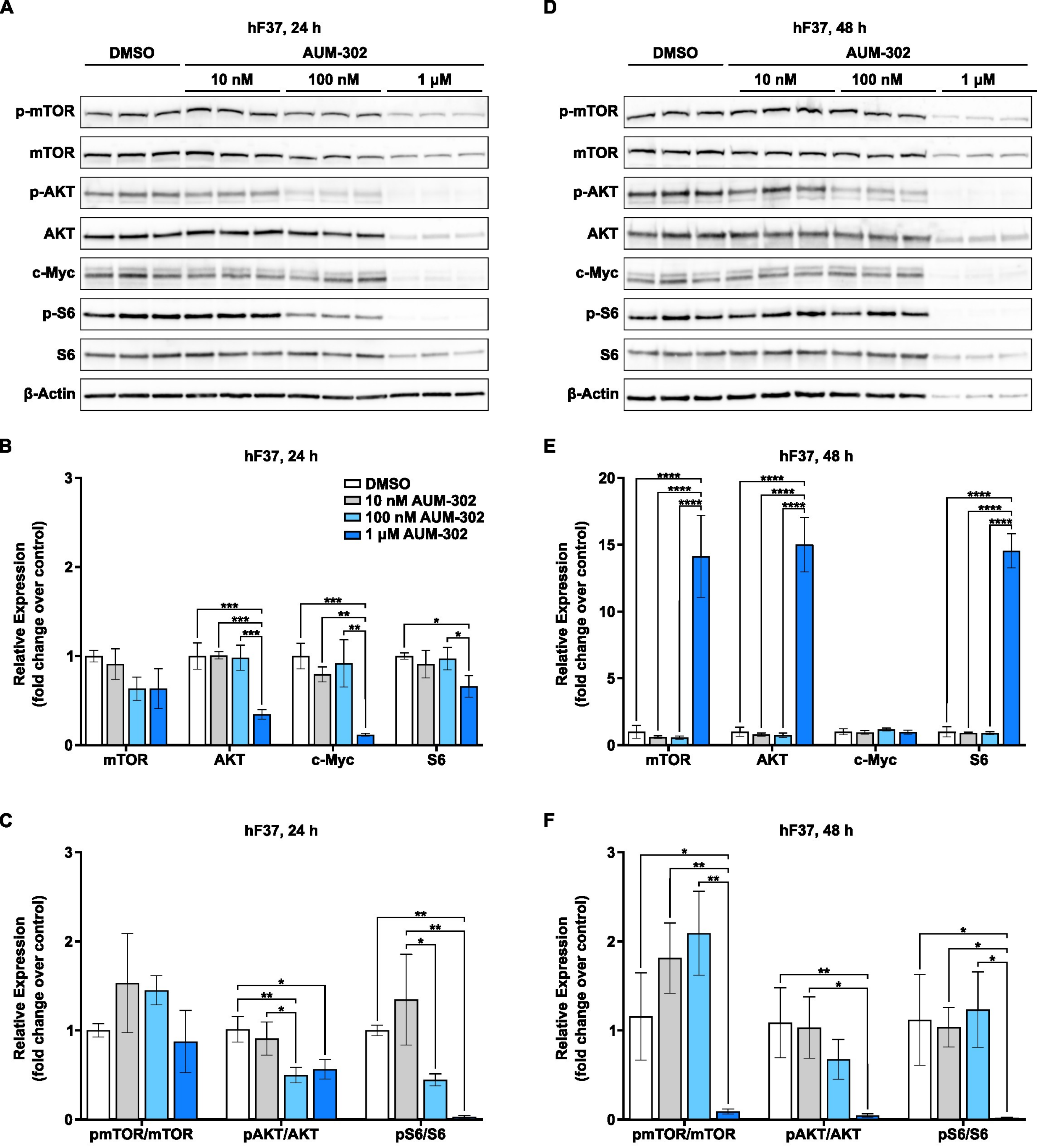

Supplementary Figure 19.

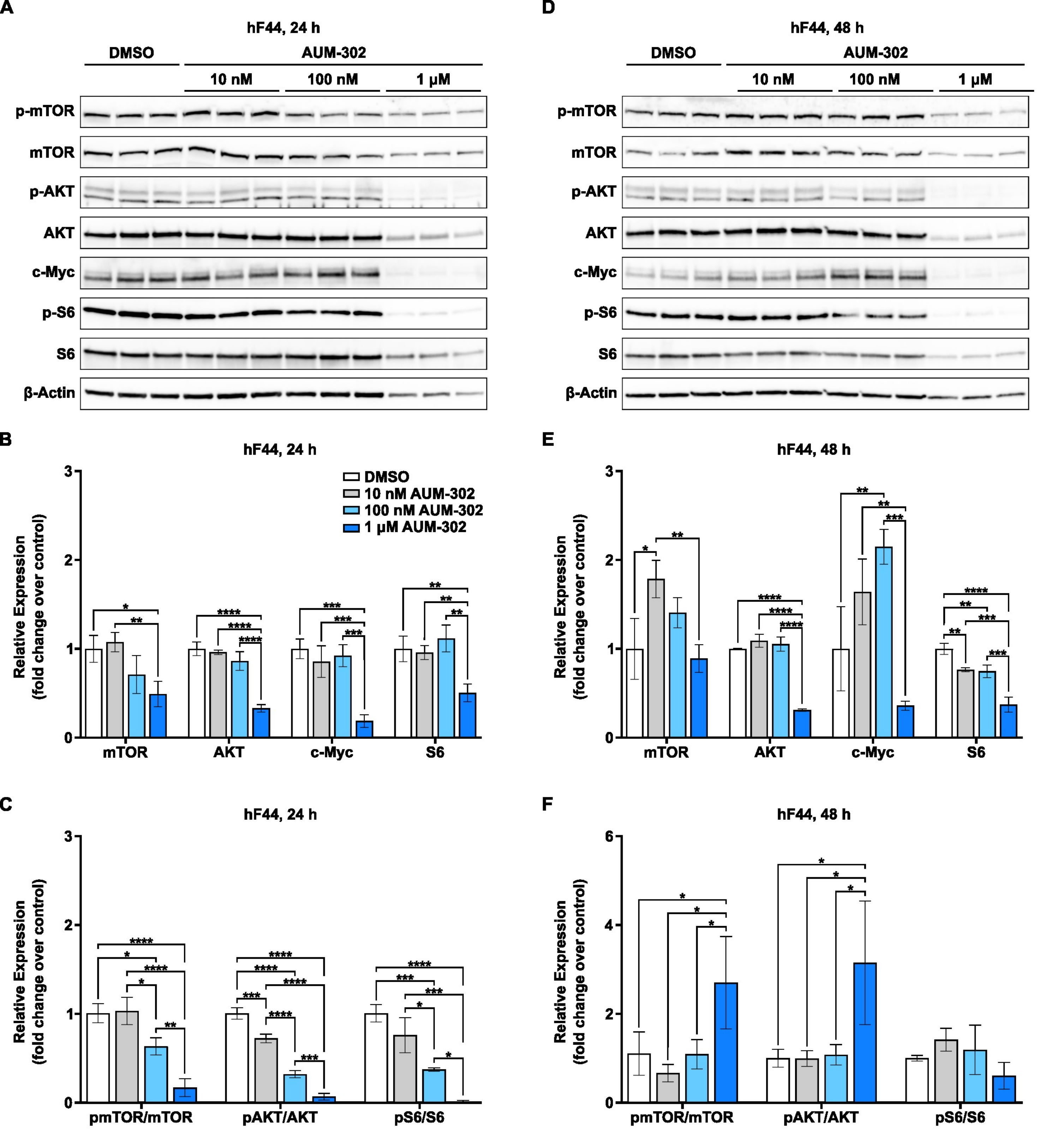

Supplementary Figure 20.

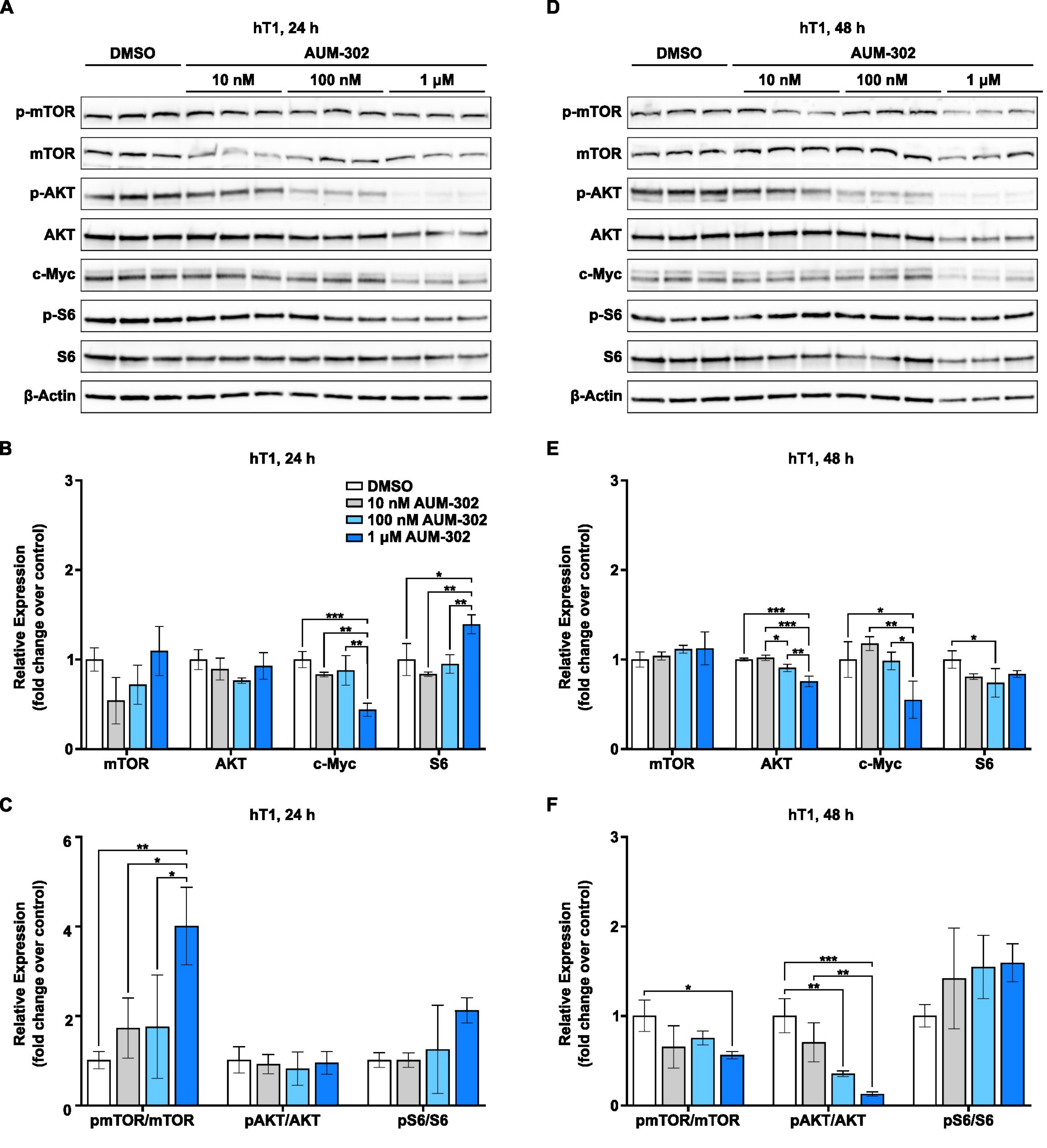

Supplementary Figure 21.

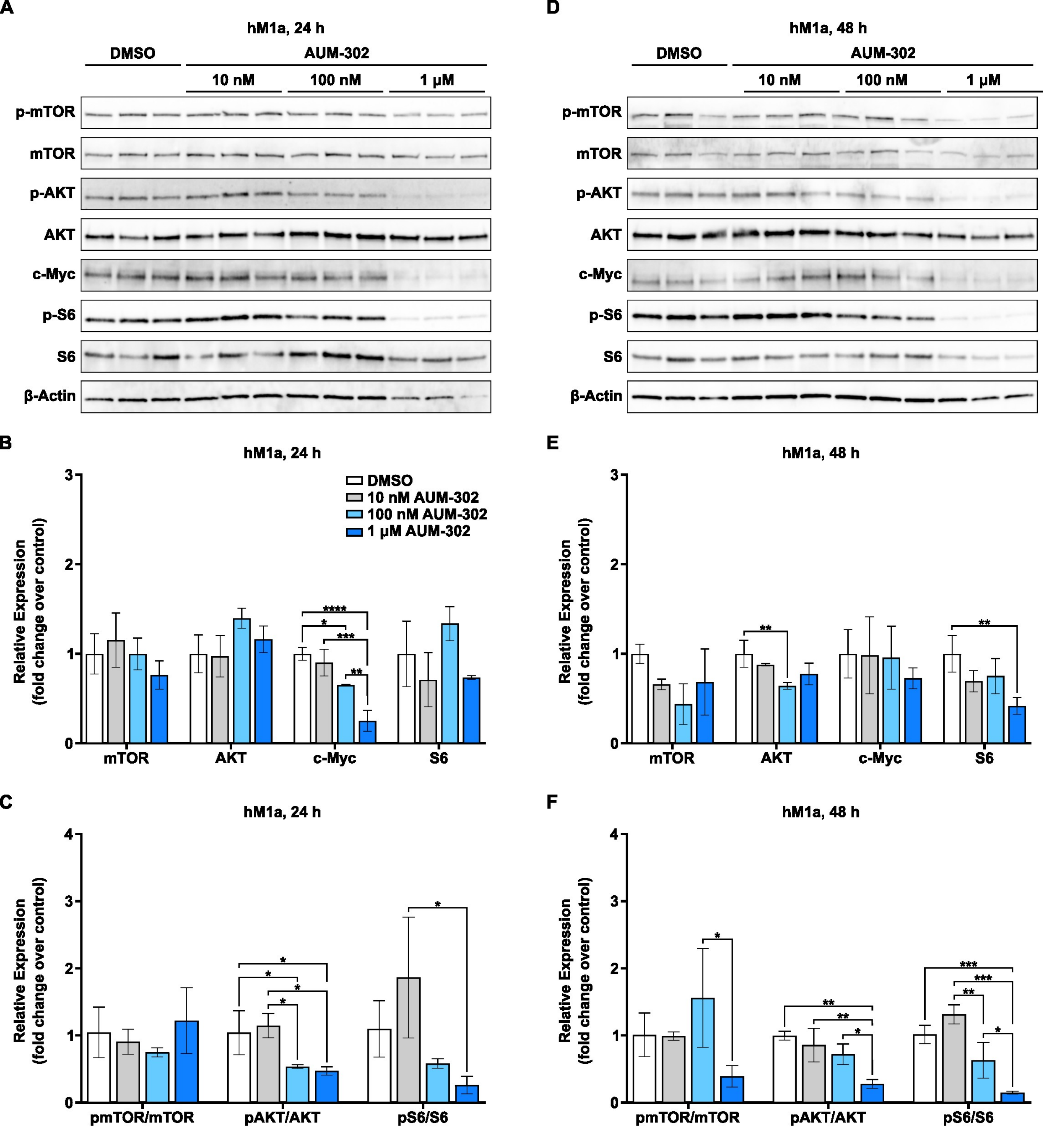

Supplementary Figure 22.

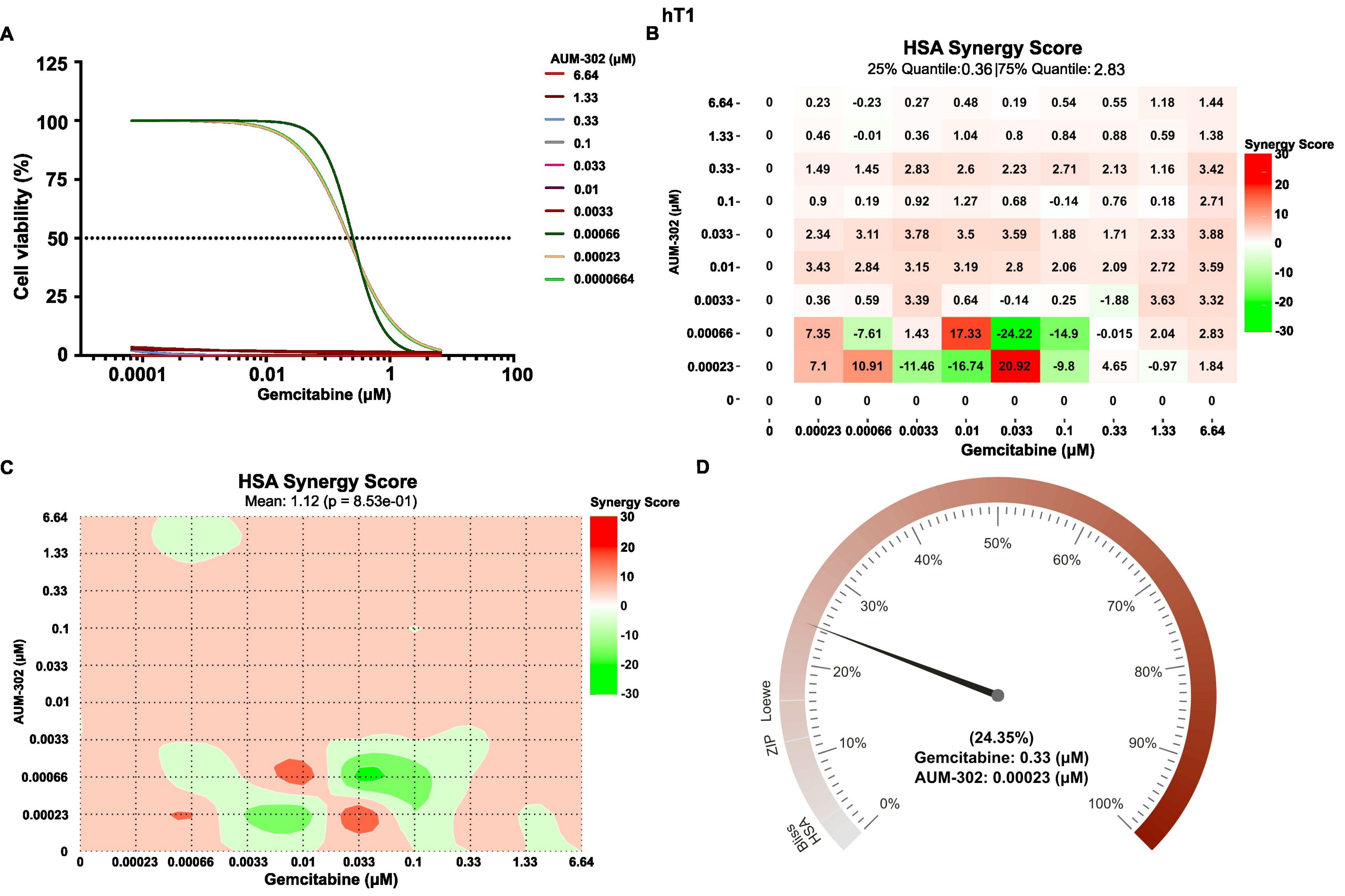

Supplementary Figure 23.

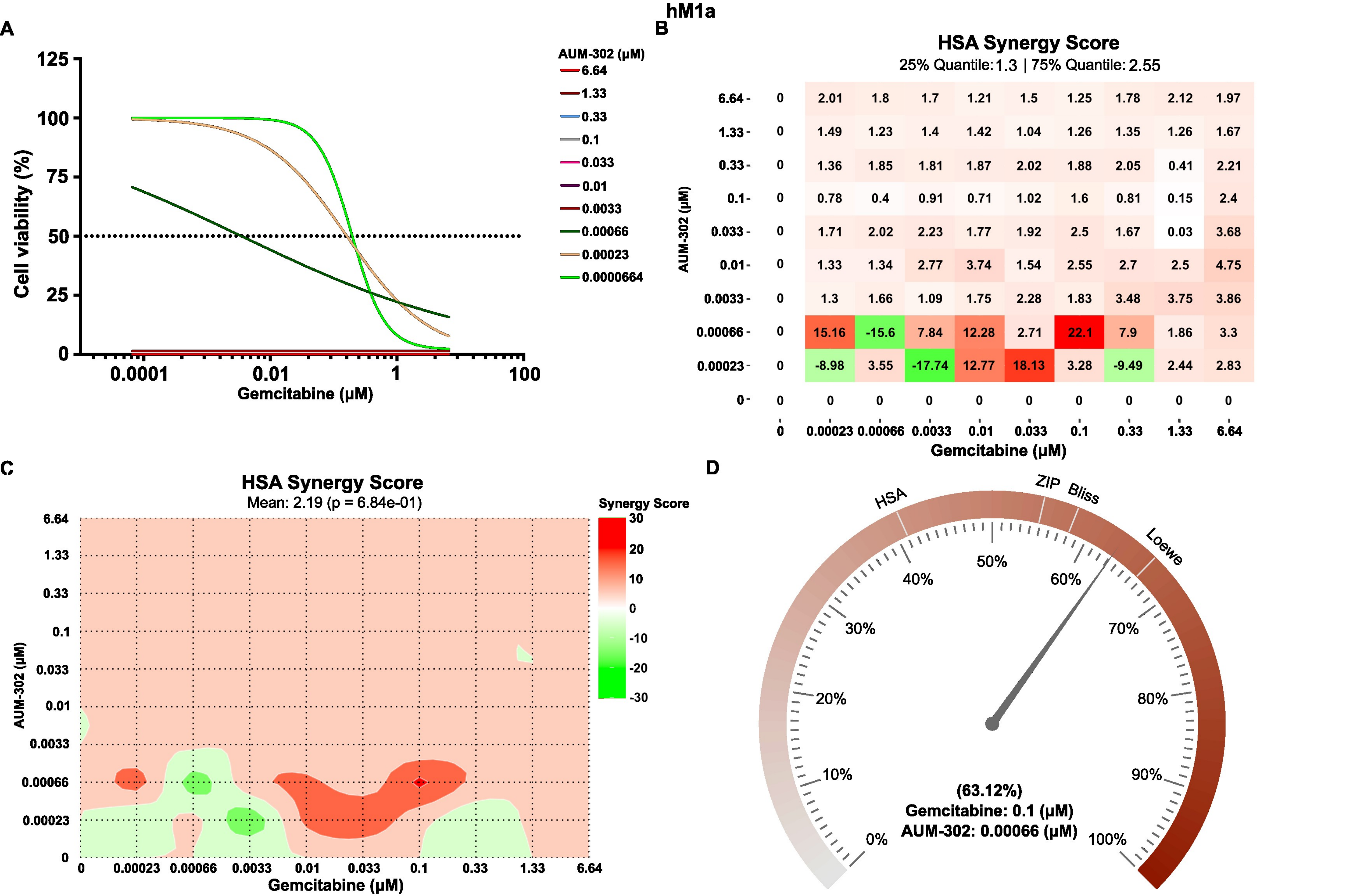

Supplementary Figure 24.

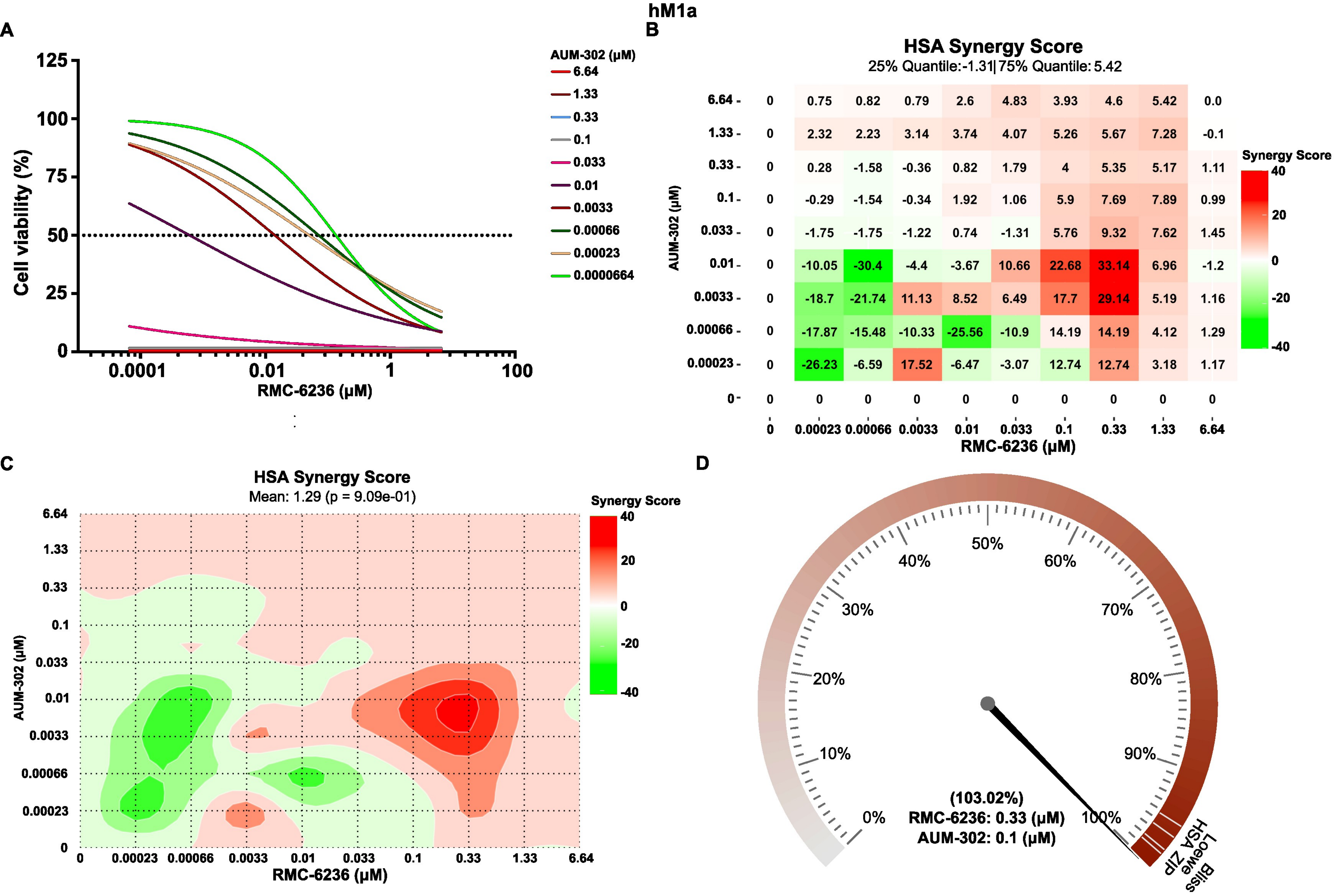

Supplementary Figure 25.

A

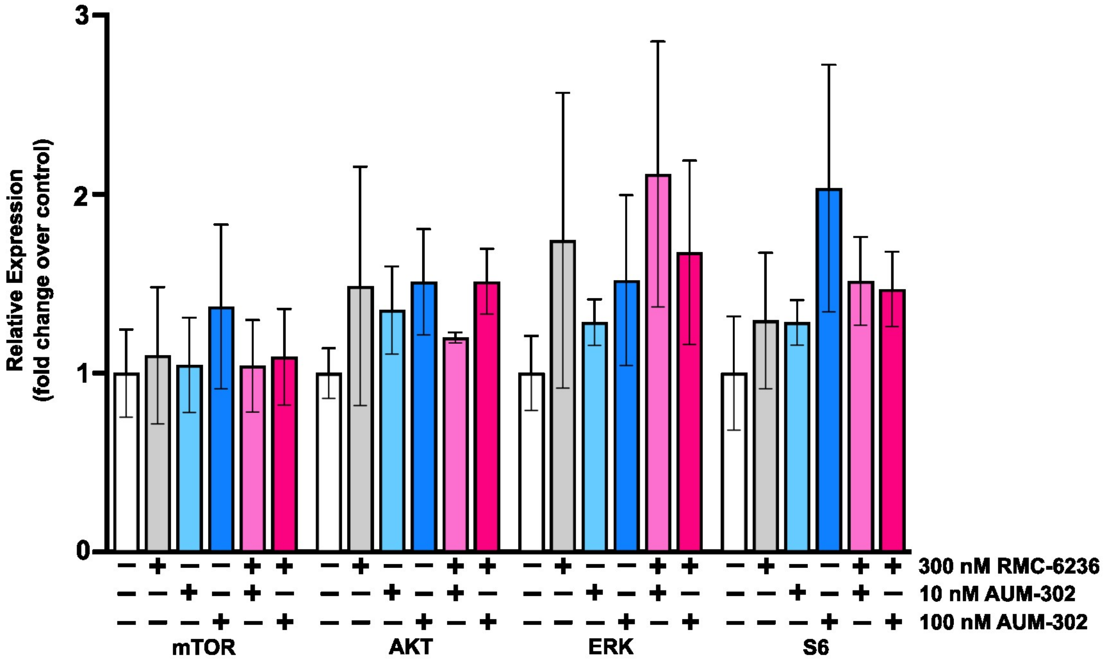

B

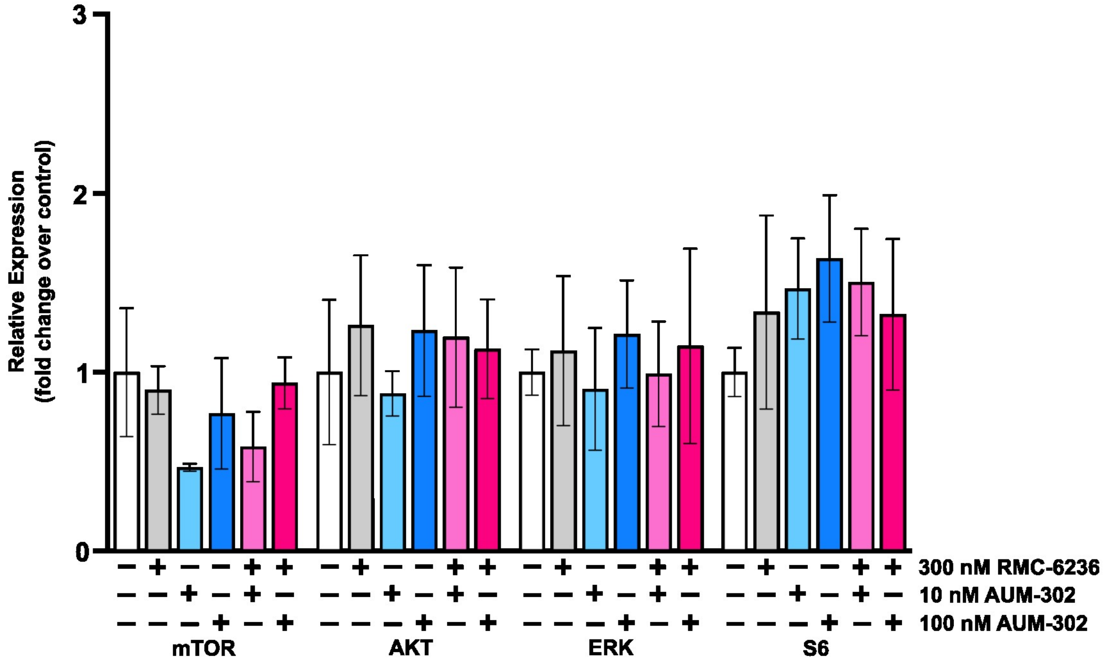

Supplementary Figure 26.
